# Age‐Related Oxidative Stress and Mitochondrial Dysfunction in Lymph Node Stromal Cells Limit the Peripheral T Cell Homeostatic Maintenance and Function

**DOI:** 10.1111/acel.70100

**Published:** 2025-05-21

**Authors:** Sandip Ashok Sonar, Ruchika Bhat, Heather L. Thompson, Christopher P. Coplen, Jennifer L. Uhrlaub, Mladen Jergovic, Janko Ž. Nikolich

**Affiliations:** ^1^ Department of Immunobiology University of Arizona College of Medicine‐Tucson Tucson Arizona USA; ^2^ The University of Arizona Center on Aging, University of Arizona College of Medicine‐Tucson Tucson Arizona USA; ^3^ BIO5 Institute, University of Arizona Tucson Arizona USA; ^4^ The Aegis Consortium for Pandemic‐Free Future University of Arizona Health Sciences Tucson Arizona USA

**Keywords:** aging, lymph node stromal cells, mitochondrial dysfunction, oxidative stress, T cell homeostasis

## Abstract

Lymph nodes (LN) are the key organs in charge of long‐term maintenance of naïve lymphocytes and their initial, primary activation upon infection. Accumulating evidence indicates that LN stromal cells undergo degenerative changes with aging that critically impair LN function, including the generation of protective primary immune responses. The nature of these defects remains incompletely understood. We here demonstrate that age‐related LN stromal changes manifest themselves in mitochondrial dysfunction and oxidative stress. Ex vivo, all three major stromal cell subsets, fibroblastic reticular cells (FRC), lymphatic endothelial cells (LEC), and blood endothelial cells (BEC) exhibit elevated mitochondrial reactive oxygen species (ROS) stress, reduced mitochondrial potential, and elevated mitochondrial mass with aging. Old FRC also exhibited elevated cytoplasmic ROS production. This was accompanied by the reduced ability of old LN stromal cells to support Tn survival in vitro, a defect alleviated by pretreating old LN stroma with the general antioxidant *N*‐acetyl cysteine (NAC) as well as by mitochondrial ROS‐reducing (mitoquinone) and mitophagy‐inducing (urolithin A) compounds. Mitochondrial dysfunction and, in particular, reduced mitochondrial potential in old FRC were also seen upon vaccination or infection in vivo. Consistent with these results, in vivo antioxidant treatment of old mice with NAC restored to adult levels the numbers of antigen‐specific CD8^+^ effector T cells and their production of granzyme B in response to antigenic challenge.

## Graphical abstract

Aging of the lymph node stromal cells limits their ability to support the survival and maintenance of peripheral naive T cells. The lymph node stromal cells are critical for providing the survival and trophic signals to peripheral naïve T cells, and after the early‐life thymic involution, these secondary lymphoid organ stromal microniches are decisive in maintaining peripheral T cells. However, our data demonstrate that age‐related changes in the lymph node stromal cells, especially increased oxidative stress and mitochondrial dysfunction, limit their ability to support the naïve T cell survival and maintenance independent of T cell age. Interestingly, we found that these age‐related defects in stromal cells are reversible, and targeting them with antioxidant (*N*‐acetyl cysteine) or mitochondrial targeting molecules (mitoquinone or urolithin A) significantly improved old lymph node stromal cell ability to support the survival of naïve T cells.

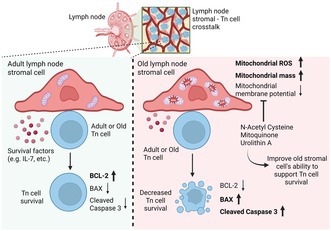

**Abbreviations**
BCL2: B‐cell lymphoma 2
BEC: blood endothelial cells
FRC: fibroblastic reticular cells
LEC: lymphatic endothelial cells
LN: lymph node
LTβ: lymphotoxin beta
LTβR: lymphotoxin beta receptor
NAC: *N*‐acetyl cysteine
Pdpn: podoplanin
pfu: plaque forming units
pLN: peripheral skin‐draining lymph nodes
ROS: reactive oxygen species
SLO: secondary lymphoid organ
tet: tetramer
Tn: naive T cells
WNV: West Nile virus

## 1 Introduction

The immune system undergoes age‐related changes that, among other consequences, result in dysfunctional immune defense which contributes to increased vulnerability to infectious diseases in older populations (Abel and Casanova 2024). Older adults are particularly susceptible to new emerging and reemerging pathogens, and often exhibit less vigorous responses to vaccination. This manifests itself by disproportionately higher morbidity and mortality among older populations during outbreaks of infections by viruses such as SARS‐CoV2, West Nile virus (WNV), and chikungunya virus as well as annual seasonal influenza, respiratory syncytial virus (RSV) and other infections (Bartoszko and Loeb 2021; Godaert et al. 2021; Kaplan and Angus 2003; Remelli et al. 2022). T cells are particularly critical in providing immune defense against intracellular infections, both by direct antiviral action and via providing help to B cells. While T cell responses in young adults are robust and effective, older adults often exhibit suboptimal T cell responses, resulting in a reduced ability to clear infection (Nikolich‐Zugich 2018). Understanding why T cell function declines with age may be a key to developing strategies to improve immune defense and resilience to infection in older adults.

Thymus involution is the earliest manifestation of immune aging. This begins well before puberty, which significantly reduces the generation of new naïve T (Tn) cells (Lynch et al. 2009) by the end of puberty and beyond. Despite this, our body maintains a relatively stable pool of Tn cells through their homeostatic maintenance in secondary lymphoid organs (SLO), most prominently in lymph nodes (LN) (Brown and Turley 2015; Krishnamurty and Turley 2020; Link et al. 2007). LN are highly organized structures that harbor both T and B lymphocytes and innate immune cells in specialized niches formed by distinct stromal cell networks. They also accept lymphatic drainage from different parts of the body, thereby continuously sampling lymph for signs of infection. Based on the surface expression of CD31 and podoplanin (Pdpn, a.k.a. gp38), LN CD45^−^ stromal fraction is classified into fibroblastic reticular cells (FRC; CD31^−^Pdpn^+^), lymphatic endothelial cells (LEC; CD31^+^Pdpn^+^), blood endothelial cells (BEC; CD31^+^Pdpn^−^), and double negative cells (DN; CD31^−^Pdpn^−^). Within each of these stromal cell types, various transcriptionally and functionally distinct subsets exist that form highly specialized niches that are absolutely required for the maintenance and function of T and B cells and APCs (Brulois et al. 2020; Kapoor et al. 2021; Rodda et al. 2018; Xiang et al. 2020).

Accumulating evidence indicates that SLO, and most notably LN, deteriorate with aging, and that this deterioration decisively contributes to the decline in both homeostatic maintenance and functional immune responsiveness (Becklund et al. 2016; Denton et al. 2022; Lee and Linterman 2022; Masters et al. 2019; Richner et al. 2015; Silva‐Cayetano et al. 2023; Sonar et al. 2022; Thompson et al. 2019). The LN from old mice appeared smaller than those from young adults and exhibited disrupted LN architecture, as evident from the loss of T cell and B cell organization and altered expression of homeostatic chemokines such as CCL19, CCL21, and CXCL13 (reviewed in Sonar et al. 2023). Becklund et al. (2016) demonstrated a decrease in the ability of old LN to support homeostatic proliferation of adoptively transferred young Tn cells. Old LN also exhibited defects in the homing and retention of new T cells generated from rejuvenated thymus and exhibited a sign of fibrosis as evident from increased accumulation of extracellular collagen in mice and nonhuman primates (Thompson et al. 2019). Such a deposition of collagens and possibly other extracellular matrix components has functional consequences on T cell homing and migration. Using a live imaging approach, Kwok et al. (2022) demonstrated that excessive collagen deposition in old LN affects the migratory behavior of naïve T cells at steady state, with T cells residing closer to fibrotic areas moving slower as opposed to those farther. Using indelible labeling and tracking of newly generated T cells in mice, we recently demonstrated that skin‐draining LN exhibit an early (at 6–9 months of life) impairment in their ability to maintain naïve T cells, which corresponded closely with alterations in FRC network and a decline in numbers of LEC (Sonar et al. 2022). Similar degenerative changes have been demonstrated in nonhuman primate and human lymph nodes (Bekkhus et al. 2023; Nakagawa et al. 2022).

In addition to their structural and homeostatic roles at steady‐state, LN stromal cells actively respond to immune challenges. The stromal cell responses are critical for the orchestration of adaptive immune responses, and genetic deletion of the stromal cell population or loss of their function results in significantly decreased T and B cell maintenance and responsiveness (Chai et al. 2013; Cremasco et al. 2014). However, old stromal cells exhibit subpar responses to infection compared to their young adult counterparts, often exemplified by reduced or delayed expansion of the draining LN, decreased homing of T cells, and reduced expansion of virus‐specific T cells, follicular helper T cells (Tfh) and germinal center (GC) B cells (Richner et al. 2015). Similar age‐related decreases in LN expansion and T cell function were noted in response to Chikungunya virus infection (Uhrlaub et al. 2016). Old LN also exhibit delayed GC formation, and GC appeared fewer and smaller, resulting in decreased antibody response to infection and vaccination (Stebegg et al. 2020). It has been demonstrated that decreased response of marginal reticular cells (MRC), a subset of FRC located beneath the sub‐capsular sinus of the LN, to model antigen underlies reduced GC B cell response and impaired antibody response in old mice, which is partly rescued by TLR4 agonists‐containing adjuvant (Denton et al. 2022). LN atrophy and loss of FRC architecture also corresponded closely with the decline in WNV‐specific CD8^+^ T cell response in old mice (Sonar et al. 2022). More recently, dysregulation of stromal cell chemokine secretion in old LN has been found to contribute to impaired localization of Tfh cells in the GC, which resulted in reduced antibody response (Silva‐Cayetano et al. 2023). Collectively, these findings strongly support the idea that the old LN stromal microenvironment adversely impacts the maintenance and function of peripheral Tn cells. However, the molecular nature of age‐related changes in LN stromal cells is not known, and it is unclear whether and to what extent they may be reversible.

Here we demonstrate that LN stromal microenvironment decisively contributes to age‐related Tn cell maintenance and to reduced antiviral T cell immunity due to increased oxidative stress and mitochondrial dysfunction in all the three major LN stromal cell subsets. Such defects were not seen in Tn cells themselves. Consistent with that finding, old LN stroma exhibited defects in supporting survival and maintenance of Tn cells regardless of Tn cell age. Furthermore, old LN stromal cells failed to expand in response to infection in vivo, resulting in subpar T cell immunity. Mechanistically, the LN stromal age‐related impairment closely coincided with mitochondrial dysfunction, characterized by higher mitochondrial mass and decreased membrane potential in stromal cells. Physiological relevance of these observations was confirmed by showing that pharmacological targeting of both oxidative stress and mitochondrial dysfunction in old LN stromal cells significantly improved their ability to support Tn cell survival and maintenance in vitro in the co‐culture model and also improved T cell function in response to WNV in vivo. Collectively, these findings point to a mechanism by which aging LN stromal microenvironment limits peripheral T cell maintenance and immune function in old mice, which is reversible and can be targeted to improve T cell maintenance and function.

## 2 Results

### 2.1 Aging of Lymph Node Stromal Cells Limits Their Ability to Maintain Naïve T Cells in a Co‐Culture System

**FIGURE 1:**
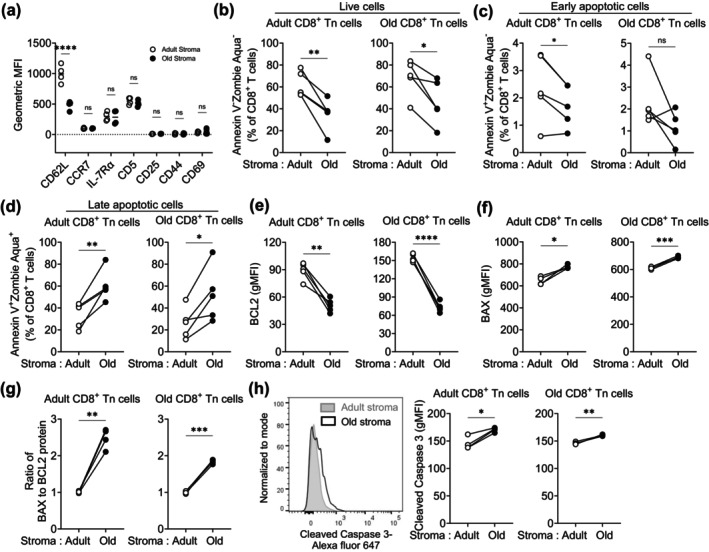
Aging of lymph node stromal cells limits peripheral T cell survival and maintenance. Purified adult and old CD8^+^CD62L^hi^CD44^lo^ naïve T cells were co‐cultured with LN stromal cells from adult and old naïve C57BL/6 mice for 4 days, and survival and maintenance of naive CD8^+^ T cells in culture was analyzed by flow cytometry. (a) Data show geometric mean fluorescence intensity (MFI) of cell surface molecules on adult naïve CD8^+^ T cells. The percentage of (b) live (Annexin‐V^−^Live/Dead‐Zombie Aqua^−^), (c) early apoptotic (Annexin‐V^+^Live/Dead‐Zombie Aqua^−^), and (d) late apoptotic (Annexin‐V^+^Live/Dead‐Zombie Aqua^+^) adult (left) and old (right) CD8^+^ T cells were shown. The geometric MFI (gMFI) of intracellular (e) BCL2 and (f) Bax protein in co‐cultured adult (left) and old (right) CD8^+^ Tn cells was shown. (g) The ratio of intracellular BAX to BCL2 proteins in co‐cultured CD8^+^ Tn cells was shown. (h) A representative histogram (left) and gMFI (right) of intracellular cleaved caspase 3 expression in adult or old CD8^+^ Tn cells co‐cultured with adult and old lymph node stromal cells were shown. Data represent measurements performed on CD8^+^ T cells co‐cultured with adult stroma (open circle) or old stroma (filled circle). Data represent 3–4 independent experiments. Two‐way ANOVA followed by Tukey's multiple comparison correction test (a), Mann–Whitney *U* test (b–h). ns, nonsignificant, *p ≤ 0.05, **p ≤ 0.01, ***p ≤ 0.001, and ****p ≤ 0.0001.

To test whether age‐related changes in LN stromal cells directly dictate the decline of peripheral T cell homeostatic maintenance, we co‐cultured adult naïve CD8^+^ T cells (CD8^+^CD62L^hi^CD44^lo^ cells; Tn in the text) with LN stromal cells purified from adult or old pLN for 4 days, and assessed stromal cells ability to support the survival of Tn cells. We used a bulk CD45^−^ stromal cell population, which contained all major stromal cell types FRC (CD31^−^Pdpn^+^), LEC (CD31^+^Pdpn^+^), BEC (CD31^+^Pdpn^−^), and DN (CD31^−^Pdpn^−^) cells in our co‐culture experiments (Figure S1a). This allowed us to study the net effect of the whole stromal compartment aging on Tn cell survival. Importantly, co‐culture conditions did not induce T cell activation, instead maintaining Tn cells in the naïve state as evident from negligible expression of T cell activation markers, CD25, CD44, and CD69, and robust expression of naïve T cell markers, CCR7, IL‐7Rα, and CD62L (Figure 1a). Further, the age of stroma did not alter the levels of these naïve state and activation markers on adult CD8^+^ Tn cells, with the exception of CD62L, whose expression was significantly decreased in the presence of old stroma as judged by mean fluorescence intensity (Figure 1a). It should be noted that enriched CD8^+^ Tn cells (purity of > 95%) had a normal range distribution of CD62L, with almost all of the cells having a higher expression of CD62L before being added to the co‐culture (Figure S1b). After 4 days of co‐culture, CD8^+^ Tn cells had a bimodal distribution of CD62L, and Tn cells co‐cultured in the presence of old stromal cells had more cells in CD62L^lo^ status than those in adult stroma, with no notable change in CD62L^hi^ cells (Figure S1b). Further, most of the Annexin‐V^+^ apoptotic CD8^+^ Tn cells had a lower surface expression of CD62L, indicating their increased vulnerability to apoptotic cell death compared to those that maintained a higher level of CD62L (Figure S1c). Moreover, the Annexin‐V^+^ status of CD62L^hi^ cells was unchanged on Tn cells co‐cultured with old or adult stromal cells. Therefore, it is unlikely that decreased CD62L expression resulted from differential survival of CD62L^lo^ cells in the presence of old stromal cells.

Old LN stromal cells, however, exhibited a significantly reduced ability to support the survival of both adult and old CD8^+^ Tn cells, and in the presence of old LN stroma cells, Tn cells exhibited increased apoptosis (Figure 1b–d). Most of the expiring Tn cells were in late apoptosis, as judged by positive staining for both Annexin V and Live/Dead, after 4 days, with fewer being in early apoptosis (Annexin V^+^ but do not stain for Live/Dead dye), or necrosis (Annexin V^−^ but stain for Live/Dead dye) (Figure S1d). Tn co‐cultured with the old stromal cells exhibited significantly more late apoptosis compared to those cultured with the adult stroma (Figure 1d). Consistent with their increased apoptosis, naïve T cells co‐cultured with old LN stromal cells exhibited reduced expression of the pro‐survival factor, B cell lymphoma 2 (BCL‐2) (Figure 1e). The role of BCL‐2 in T cell survival is extensively documented. Using genetic models, it has been conclusively demonstrated that BCL‐2 deficient Tn cells do not survive well (Nakayama et al. 1993), while BCL‐2 overexpressing Tn cells delay apoptotic cell death and survive longer (Sentman et al. 1991; Strasser et al. 1991). In addition to decreased BCL‐2, we noted an increased expression of pro‐apoptotic molecules, BCL‐2‐associated X protein (BAX) in CD8^+^ Tn cells co‐cultured with old lymph node stromal cells (Figure 1f). Upon activation by apoptosis‐inducing signals, BAX, in addition to other antagonists of BCL‐2 protein, oligomerizes in the mitochondrial outer membrane, forming pores leading to an increase in the permeabilization of the mitochondrial membrane and initiates the apoptotic cell death pathway. The BCL‐2 is a potent antagonist of BAX, which prevents BAX oligomerization and inhibits cell death by apoptosis. However, simultaneous decrease in BCL‐2 and increase in BAX levels can increase the ratio of BAX to BCL‐2 proteins in a cell. The higher BAX to BCL‐2 ratio is known to promote apoptosis in a wide variety of cell types (Del Principe et al. 2016; Schroer et al. 2023). We found Tn cells co‐cultured with old lymph node stromal cells not only had a significantly increased ratio of BAX to BCL‐2 (Figure 1g) but also increased levels of caspase 3 activation (Figure 1h).

The reduced ability of old lymph node stromal cells to support the survival of Tn cells might be due to the lack of positive (survival) signals or the provision of negative (cell death) signals by old stromal cells to Tn cells. To understand this, we co‐cultured adult Tn cells with a monolayer prepared from different ratios of adult and old stromal cells. The presence of increasing numbers of adult stromal cells in a mixed monolayer of adult/old stromal cells was able to increase the survival of Tn cells (Figure S2a) and curbed the apoptosis (Figure S2b,c). Furthermore, Tn cells co‐cultured with increasing numbers of adult cells in a mixed monolayer did exhibit a significant increase in BCL‐2 level (Figure S2d) and decline in BAX to BCL2 ratio (Figure S2e) and associated reduction in activation of caspase 3 (Figure S2f). These results suggest that adult stromal cells might be able to provide positive (survival) signals, compensating for their lack or underproduction by old stromal cells. To test this possibility, we provided purified recombinant IL‐7, a T cell survival cytokine, in the co‐culture after seeding Tn cells. Within the lymph node, FRC and LEC produce IL‐7 and support Tn survival (Onder et al. 2012), and genetic deficiency of IL‐7 in mice or anti‐IL‐7Rα treatment of wild‐type mice led to poor survival of CD8^+^ Tn cells (Tan et al. 2001). The IL‐7 promotes the T cell survival via multiple mechanisms by supporting metabolic programs required for cell survival and also regulating the expression of survival molecules, such as BCL‐2, BCL‐xL, and MCL‐1, via STAT5 and PI3K pathways and inhibiting pro‐apoptotic molecules involved in the mitochondrial apoptosis pathway (Jacobs et al. 2010). The exogenous provision of recombinant IL‐7 rescued these Tn cells from undergoing increased apoptosis in the presence of old stromal cells (Figure S1e,f). Interestingly, the survival of adult and old CD8^+^ Tn cells was comparable when co‐cultured with the adult LN stromal cells (Figure 1b–d), indicating that the age of stromal cells, and not of a Tn cell, is the primary limiting factor for Tn cell survival.

### 2.2 Old LN Stromal Cells Exhibit Oxidative Stress and Mitochondrial Dysfunction

**FIGURE 2:**
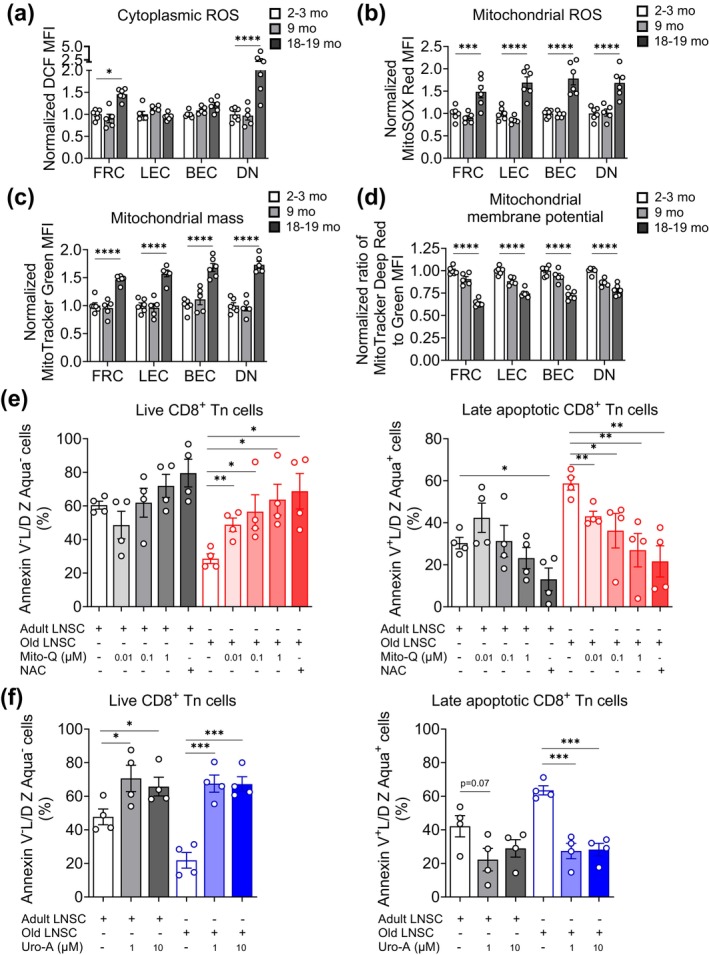
Old LN stromal cells experience oxidative stress and exhibit mitochondrial dysfunction. Peripheral LN (pooled inguinal, axillary and brachial LN) were digested with Liberase‐TL and DNase‐I and single cells suspension were treated with (a) 2′,7′‐dichlorofluorescin‐diacetate (DCF‐DA; 1 μM) for 15 min, (b) MitoSOX‐Red (5 μM) for 30 min, (c, d) MitoTracker Green‐FM (200 nM) and MitoTracker Deep Red‐FM (200 nM) for 30 min at 37°C in 5% CO_2_ incubator. Cells were washed and stained for surface markers to identify stromal cell populations and analyzed by flow cytometry. Data show level of (a) cellular (cytoplasmic) ROS, (b) mitochondrial ROS, (c) mitochondrial mass, and (d) mitochondrial membrane potential in LN stromal cell subsets, FRC (CD45^−^Pdpn^+^CD31^−^), LEC (CD45^−^Pdpn^+^CD31^+^), BEC (CD45^−^Pdpn^−^CD31^+^), and double negative (CD45^−^Pdpn^−^CD31^−^) cells from young adult (2–3 months), mid‐age (9 months), and old (18–19 months) mice. Data represent normalized MFI of the indicated parameters relative to 2–3 months of the corresponding cell population. Each dot represents an individual mouse. (e, f) Adult and old lymph node stromal cells were treated with the indicated concentrations of (e) Mitoquinone (Mito‐Q) or *N*‐acetyl cysteine (NAC; 1 mM) and (f) urolithin‐A (Uro‐A) for 24 h, cultures were washed, and purified adult CD8^+^ Tn cells were co‐cultured with treated and untreated stromal cells for 4 days. (e) Data show Annexin‐V^−^Live/Dead^−^ live (left) and Annexin‐V^+^Live/Dead^+^ late apoptotic (right) CD8^+^ Tn cells co‐cultured with Mito‐Q or NAC treated stromal cells. (f) Data show Annexin‐V^−^Live/Dead^−^ live (left) and Annexin‐V^+^Live/Dead^+^ late apoptotic (right) CD8^+^ Tn cells co‐cultured with Uro‐A treated stromal cells. Data are representative of four (a–d) or two (e, f) independent experiments and expressed as mean ± SEM. ANOVA followed by Tukey's multiple comparison correction test (a–d), Unpaired *t*‐test (e, f). ns, nonsignificant; *p ≤ 0.05, **p ≤ 0.01, ***p ≤ 0.001, and ****p ≤ 0.0001.

To investigate why old LN stromal cells failed to support the survival of peripheral Tn cells, we analyzed whether these cells may exhibit stereotypical mesenchymal age‐related defects such as mitochondrial dysfunction, which is one of the key hallmarks of aging (Li et al. 2024). Since perturbations in mitochondrial homeostasis are often linked with increased oxidative stress, we first measured intracellular reactive oxygen species (ROS) levels in LN stromal cells from young adult (2–3 months), mid‐age (9 months), and old (18–19 months) mice. We used dichlorodihydrofluorescein diacetate, a cell permeable ester that is hydrolyzed by cytoplasmic esterases, and is subsequently oxidized by various ROS to generate fluorescent 2′,7′‐dichlorofluorescein (DCF; Wardman 2007). The ex vivo isolated LN stromal cells from old mice showed significantly increased cellular ROS levels compared to cells from the young adult group (Figure S3a). Subset analysis demonstrated that old FRC and DN cells, but not LEC and BEC, exhibited increased cytoplasmic ROS (Figure 2a). More importantly, all main stromal cell types, including LEC and BEC, exhibited increased mitochondrial oxidative stress with aging, as measured by increased levels of fluorescent oxidized MitoSOX‐Red (Figure 2b; Figure S3b). Mitochondrial dysfunction is closely linked to cellular senescence and aging phenotypes. It is typically defined by the presence of three key defects: a decrease in respiratory capacity per mitochondrion, decreased mitochondrial membrane potential, and increased mitochondrial ROS levels (Miwa et al. 2022). Old LN stromal cells, including FRC, LEC, and BEC, but not the ones from the mid‐age group, clearly exhibited increased mitochondrial mass compared to their young adult counterparts (Figure S3c; Figure 2c). Despite having larger mitochondria (as a result of increased mass), old LN stromal cells also exhibited significantly reduced mitochondrial membrane potential, as measured by the ratio of fluorescence derived from mitochondrial membrane potential dependent (MitoTracker Deep Red) and independent (MitoTracker Green‐FM) probes, compared to cells from the adult group (Figure S3d; Figure 2d). The decreased mitochondrial membrane potential in old FRC, LEC, and BEC indicates the reduced mitochondrial respiratory capacity, as reduced membrane potential is directly linked to the reduced proton gradient across the inner and outer mitochondrial membranes and reduced ATP synthesis (Zorova et al. 2018). To test if the mitochondrial dysfunction in the old lymph node stromal cells affects their ability to support the survival and maintenance of Tn cells, we pretreated stromal cells with compounds known to improve mitochondrial homeostasis and function. Since old lymph node stromal cells exhibited elevated production of mitochondrial ROS, we treated stromal cells with mitoquinone (Mito‐Q). Mito‐Q is a mitochondrial‐targeted antioxidant that neutralizes mitochondrial ROS and promotes mitochondrial homeostasis and function (Fiorenza et al. 2024; Williamson et al. 2020). Mito‐Q‐treated stromal cells exhibited improvement in their ability to support the survival of Tn cells in a dose‐dependent manner (Figure 2e; Figure S3e). The improvement is more evident in old stromal cells than in adult cells (Figure 2e). Similarly, we treated stromal cells with urolithin‐A (Uro‐A), a gut microbial metabolite, which is a potent inducer of mitophagy response (D'Amico et al. 2021; Luan et al. 2021; Ryu et al. 2016). Mitophagy is a highly regulated physiological response that selectively breaks down and recycles damaged and dysfunctional mitochondria to promote mitochondrial homeostasis, cellular bioenergetics, and function under various stress conditions. Pretreatment of stromal cells with Uro‐A before culture with Tn cells enhanced their ability to support the survival of Tn cells and significantly reduced the apoptosis of Tn cells (Figure 2f; Figure S3f). Collectively, these results indicate that old LN stromal cells exhibit oxidative stress and mitochondrial dysfunction, which reduces their ability to support the naïve T cell survival and maintenance, but that only 24 h of pretreatment with general or mitochondria‐specific antioxidants or by inducers of mitophagy all improve this supportive function in LN stroma.

### 2.3 Mitigating Lymph Node Stromal Cell Oxidative Stress or the Deficiency in LTβ In Vitro Improves the Survival of Naïve T Cells

In parallel, we performed a preliminary analysis of transcriptional LN profiles using miniarrays, and found that old LN exhibited decreased mRNA expression of several tumor necrosis factor superfamily molecules, including TNFSF‐10, TNFSF‐11, TNF, and lymphotoxin‐beta (LTβ) (Figure S4). Of these, LTβR is known to be essential for the development and maturation of LN stromal structures, and genetic deletion of LTβR within FRC impaired the expression of IL‐7 and of homeostatic chemokines CCL19 and CCL21 (Chai et al. 2013). Within the LN, dendritic cells, T cells, and ILC3s produce lymphotoxin‐alpha (LTα) and ‐beta (LTβ) and engaging of heterotrimeric LTα_1_β_2_ or activated T cell‐derived LIGHT with LTβR on LN stromal cells stimulates stromal cell survival, proliferation, and production of chemokine and cell adhesion molecules.

**FIGURE 3:**
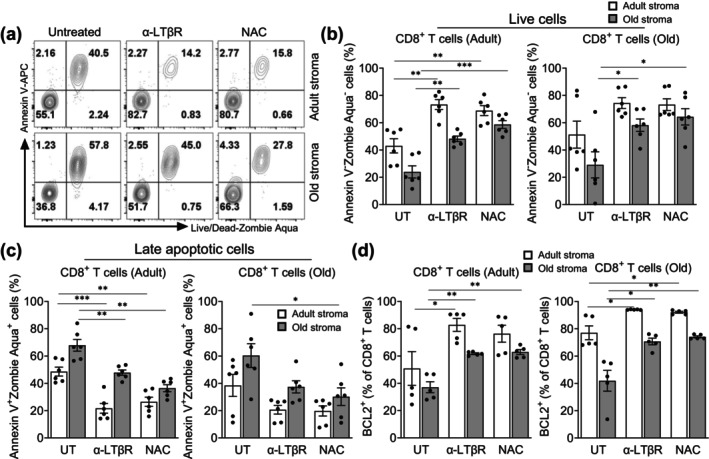
Mitigating lymph node stromal cell oxidative stress improves the survival of naïve T cells. Purified pLN stromal cells from adult and old naïve C57BL/6 mice were cultured overnight and treated with *N*‐acetyl cysteine (NAC, 1 mM) or anti‐LTβR (2 μg/mL) for 24 h. The next day, purified CD8^+^CD62L^hi^CD44^lo^ naïve T cells from adult and old C57BL/6 mice were co‐cultured with stromal cells for 4 days, and survival of CD8^+^ T cells in culture was analyzed by flow cytometry. (a) Representative flow cytometry plots (gated on CD8^+^ T cells) show staining of Annexin‐V and Live/Dead‐Zombie Aqua. Numbers in the quadrant indicate the percentage of cells. (b) Percentage of live (Annexin‐V^−^Live/Dead‐Zombie Aqua^−^) and (c) apoptotic (Annexin‐V^+^Live/Dead‐Zombie Aqua^+^) adult (left) and old (right) CD8^+^ T cells co‐cultured with either adult stroma (open bars) or old stroma (filled bars) were shown. (d) Data show the percentage of intracellular Bcl2^+^ cells within the CD8^+^ T cell population. Data pooled from 3 to 4 independent experiments and expressed as mean ± SEM. Each dot represents the mean of duplicate measurements performed in each experiment. Two‐way ANOVA followed by Tukey's multiple comparison correction test. ns, nonsignificant; *p ≤ 0.05, **p ≤ 0.01, ***p ≤ 0.001.

Based on the above results, we sought to examine whether reducing age‐related oxidative stress via *N*‐acetyl cysteine (NAC), an antioxidant known to mitigate elevated ROS, or providing agonistic anti‐LTβ receptor (α‐LTβR) signaling (Kumar et al. 2015), may improve the function of old LN stromal cells in vitro. We found that both the agonistic α‐LTβR and NAC treatment of adherent stromal cells for 24 h prior to the addition of Tn cells in the co‐cultures led to significant improvement in cocultured Tn cell survival (Figure 3a,b), with significant reduction of CD8^+^ Tn apoptotic death (Figure 3c) and augmented expression of the pro‐survival BCL2 molecule (Figure 3d), regardless of the age of CD8^+^ Tn cells. Moreover, old stroma treatment with α‐LTβR or NAC led to restoration of surface levels of CD62L comparable to that seen with Tn cells co‐cultured with untreated adult stroma (Figure S5). These data suggest that age‐related changes in LN stromal cells are reversible, and that approaches to mitigate age‐related increases in oxidative stress and decreases in LTβ signaling can improve their ability to support the survival and maintenance of peripheral Tn cells.

### 2.4 Naïve T Cells Are Poorly Maintained In Situ in Old Lymph Nodes

Since our co‐culture experiments revealed that old LN stromal cells poorly support the survival of CD8^+^ Tn cells, we analyzed T cell survival in the murine old LN using Annexin V and fixable live/dead staining. Our analysis showed that at steady state about 1%–2% CD4^+^ (2.04 ± 0.42) and CD8^+^ (0.99 ± 0.1) T cells were annexin V‐positive but did not stain for Live/Dead stain, indicative of a low level of early apoptosis in adult LN, whereas these proportions significantly increased in old LN (6.05 ± 1.19, CD4^+^ T cells; 2.83 ± 1.65, CD8^+^ T cells) (Figure S6a,b). A similar fraction of adult CD4^+^ (2.15 ± 0.35) and CD8^+^ (1.47 ± 0.46) T cells positively stained with both Annexin V and Live/Dead stain, indicating a late‐apoptotic state, and these proportions were also significantly increased in old LN (6.30 ± 1.98, CD4^+^ T cells; 3.43 ± 2.13, CD8^+^ T cells). Consistent with the increased apoptosis, the CD4^+^ and CD8^+^ T cells in the old LN exhibited increased levels of cleaved caspase3, indicative of an active apoptotic cell death program, as compared to cells from the adult LN (Figure S6c). To assess whether the old lymph node stromal microenvironment may actively contribute to the increased apoptosis of Tn cells by secreting pro‐apoptotic molecules, we cultured CD4^+^ and CD8^+^ Tn cells from adult and old mice with or without culture supernatant derived from adult or old lymph node stromal cells and analyzed their survival by flow cytometry. Adult CD4^+^ and CD8^+^ Tn cells did not survive well in the presence of culture supernatant derived from old lymph node stromal cells compared to the supernatant from adult stromal cells (Figure S6d), with adult CD8^+^ Tn cells surviving worse than the adult CD4^+^ Tn cells (Figure S6d). By contrast, the survival of old CD4^+^ and CD8^+^ Tn cells was comparable in the presence of supernatants from either adult or old stromal cells (Figure S6d–f).

### 2.5 Oxidative Stress and Mitochondrial Dysfunction in Old Lymph Node Stromal Cells in the Course of an Immune Response to WNV Infection

Since old LN stromal cells exhibited elevated oxidative stress and mitochondrial dysfunction and were unable to support and maintain naïve T cells, we sought to examine the age‐related changes in stromal cell response to infections. We infected adult and old C57BL/6 mice with WNV via footpad and analyzed the responses of T cells and stromal cells in the draining popliteal and inguinal LNs. We used H2‐D^b^:NS4b_2488–2496_ tetramer (NS4b‐tet) to track CD8^+^ T cells reactive to WNV immunodominant peptide. Consistent with prior reports (Richner et al. 2015), we observed reduced numbers of NS4b‐tet^+^CD8^+^ T cells in draining LN (dLN) of old mice compared to adult ones (Figure S7a) and concomitantly reduced numbers of circulating NS4b‐tet^+^CD8^+^ T cells in the old mice (Figure S7b). The LN typically responds to infection or inflammation by lymphadenopathy, an expansion of LN volume to accommodate increasing numbers of immune cells (resident plus recirculating cells and their proliferative burst). This is primarily driven by stromal cell responses that include activation, stretching, and proliferation. In line with the observed reduction in WNV‐specific T cell response, unlike adult LN, the old LN failed to increase their total cellularity, indicating less space available to recruit immune cells to the response (Figure S7c). This is exemplified by fewer CD45^−^ stromal cells found within old LN compared to adult LN, suggesting stromal cells did not proliferate/expand during the infection (Figure S7d). Among the major stromal cell subsets, the FRC showed a striking difference in their response to WNV. As expected, adult FRC responded promptly to the infection challenge with an increased number of FRC in adult LN; however, old FRC failed to mount a similar response (Figure S7e). The LEC and DN cells followed a similar trend but failed to reach statistical significance (Figure S7e).

**FIGURE 4:**
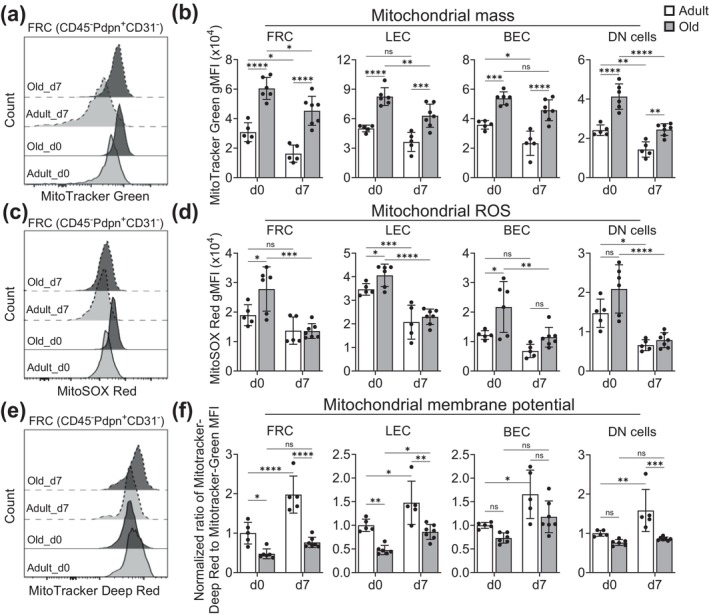
Oxidative stress and mitochondrial dysfunction in old lymph node stromal cells limit their response to West Nile virus infection. Adult (2–4 months) and old (18–20 months) C57BL/6 mice were infected with WNV via s.c. in hind foot‐pads. At day 7, draining LN were harvested and digested to isolated stromal cells. As in Figure 2, mitochondrial parameters were analyzed in LN stromal cells. Representative flow cytometry histograms show staining profile for (a) MitoTracker Green indicating mitochondrial mass, (c) MitoSOX Red indicating mitochondrial ROS, and (e) MitoTracker Deep Red indicating membrane potential‐dependent mitochondrial mass within FRC (CD45^−^Pdpn^+^CD31^−^) population at 0 and 7 dpi. The corresponding group level comparison of (b) mitochondrial mass, (d) mitochondrial ROS, and (f) membrane potential in FRC (CD45^−^Pdpn^+^CD31^−^), LEC (CD45^−^Pdpn^+^CD31^+^), BEC (CD45^−^Pdpn^−^CD31^+^), and DN (CD45^−^Pdpn^−^CD31^+^) population were depicted. Each dot represents an individual mouse. Data are representative of three independent experiments and expressed as mean ± SEM. Each dot represents an individual mouse. Two‐way ANOVA followed by Tukey's multiple comparison correction test (b, d, f). ns, nonsignificant; *p ≤ 0.05, **p ≤ 0.01, ***p ≤ 0.001, and ****p ≤ 0.0001.

To test if elevated oxidative stress and mitochondrial dysfunction in the stromal cell compartment contribute to the observed decline in response to infection in old mice, we challenged adult and old B6 mice with WNV and analyzed mitochondrial mass, membrane potential, and ROS in LN stromal cells and lymphocytes at day 0 and 7. All major stromal cell types exhibited mitochondrial defects, as judged by increased mitochondrial mass, elevated ROS, and reduced mitochondrial membrane potential at steady state (Figure 4a–f). Adult FRC, LEC, BEC, and DN cells responded well to WNV infection by augmenting their mitochondrial membrane potential and lowering ROS levels and mitochondrial mass, suggestive of an active proliferative response (Figure 4b,d,f). By day 7, although adult and old FRC, LEC, BEC, and DN cells had comparable ROS levels (Figure 4d), old stromal cells exhibited higher mitochondrial mass, suggestive of more fused and elongated mitochondria (Figure 4b). Despite the fact that old stromal cell subsets exhibited a higher mitochondrial mass, they failed to augment their mitochondrial membrane potential to the level achieved by their adult counterparts (Figure 4f), indicating that their mitochondria were largely in a depolarized state and not functionally comparable to adult stromal cells.

Tn cell differentiation into effector/memory cells in response to antigenic stimulation is closely associated with changes in mitochondrial structure and dynamics, ensuring highly proliferating cells efficiently adapt to meet their bioenergetic demand via metabolic reprogramming. Naïve T cells carry elongated and larger mitochondria while effector T cells display fragmented mitochondria and often exhibit reduced mitochondrial mass and ROS (Corrado and Pearce 2022). Consistent with this, a striking reduction in mitochondrial mass (Figure S8a) and ROS levels (Figure S8b) and increased mitochondrial membrane potential (Figure S8c) were observed in adult NS4b‐tet^+^CD8^+^ T cells at day 7 postinfection compared to the day 0 state. At steady state, the old NS4b‐tet^+^CD8^+^ T cells had increased mitochondrial ROS levels but comparable mitochondrial mass and membrane potential compared to their adult counterparts (Figure S8a–c), indicating an absence of overt mitochondrial issues. Old NS4b‐tet^+^CD8^+^ T cells also followed a similar trend as their adult counterparts and exhibited significantly decreased mitochondrial mass and ROS levels and a modest boost in mitochondrial membrane potential on day 7 postinfection compared to day 0. However, these mitochondrial parameters in old cells failed to reach the levels of adult NS4b‐tet^+^CD8^+^ T cells (Figure S8a,c), implying that old antigen‐specific T cells tend to possess more depolarized or otherwise defective mitochondria than adult T cells. Such accumulation of defective mitochondria in old antigen‐specific effector T cells can limit their functionality, as previously seen within the tumor microenvironment (Yu et al. 2020), wherein T cells with depolarized mitochondria were reported to exhibit exhaustion signature and reduced anti‐tumor function. However, this might be a result of sampling a fewer antigen‐specific cells at day 0 owing to the fact that we had found on average 2‐ to 3‐fold more NS4b‐tet^+^CD8^+^ precursor T cells in LN at day 0 or likely difference in the kinetics of NS4b‐tet^+^CD8^+^ T cell response in old LN relative to the adult LN. To better understand if this issue is evident in old naïve T cells (equivalent to NS4b‐tet^+^CD8^+^ precursor T cells) in steady‐state LN, we analyzed mitochondrial parameters in naïve (CD62L^hi^CD44^lo^) phenotype CD8^+^ and CD4^+^ T cells. Our analysis showed that old naïve CD8^+^ T cells had a modestly higher mitochondrial mass but equivalent ROS levels and mitochondrial membrane potential than adult naïve CD8^+^ T cells (Figure S8d–f). Further, adult and old naïve CD4^+^ T cells did not show any sign of oxidative stress and mitochondrial dysfunction (Figure S8d–f), suggesting that unlike stromal cells, T cells capable of responding to new infections possess functional mitochondria in old LN.

Collectively, these data suggest that mitochondrial dysfunction in old lymph node stromal cells correlated with their failure to expand and respond to WNV infection, which may contribute to fewer NS4b‐tet^+^CD8^+^ T cells.

### 2.6 Targeting Oxidative Stress, but Not LTβ Signaling, Improved Functional T Cell Immunity to WNV Infection in Old Mice

**FIGURE 5:**
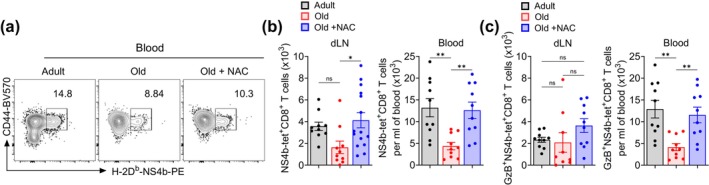
Targeting oxidative stress improved functional T cell immunity to West Nile virus infection in old mice. Old (18–20 months) C57BL/6 male mice treated with *N*‐acetyl cysteine (NAC) every alternate day by oral gavage for four continuous weeks followed by s.c. infection with WNV in both hind foot‐pads. At 7 dpi, WNV‐specific T cell response in blood and dLN was analyzed. (a) Representative flow cytometry plots (gated on live, CD3^+^CD4^−^CD8^+^ T cells) show staining of CD44 and H‐2D^b^‐NS4b‐PE tetramer in blood from untreated adult and old, and NAC‐treated old mice. (b) Absolute numbers of H‐2D^b^‐NS4b‐tetramer^+^CD8^+^ T cells in the dLN (left) and blood (right) were shown. (c) Absolute numbers of granzyme B^+^H‐2D^b^‐NS4b‐tetramer^+^CD8^+^ T cells in the dLN (left) and blood (right) were shown. Data are pooled from two independent experiments and expressed as mean ± SEM. Each dot represents an individual mouse. Numbers next to the box indicate the percentage of positive cell population (a). One‐way ANOVA followed by Tukey's multiple comparison test (b, c). ns, nonsignificant; *p ≤ 0.05, and **p ≤ 0.01(b, c).

To extend our observations from in vitro co‐culture experiments, where we observed that treatment with agonistic anti‐LTβR boosted the ability of old stromal cells to maintain Tn cells, we performed injections of agonistic anti‐LTβR antibody in old mice at days 9, 6, and 3 before being challenged with WNV. Consistent with our data (Figure 5a,b; Figure S7a,b), old mice had fewer NS4b‐tet^+^CD8^+^ T cells than adult mice (Figure S9a). However, we did not find an improvement in the numbers of NS4b‐tet^+^CD8^+^ T cells in dLN and blood in anti‐LTβR‐treated old mice compared to untreated old controls (Figure S9a). Further, anti‐LTβR treatment did not boost the Granzyme B^+^NS4b‐tet^+^CD8^+^ T cells (Figure S9b).

We then explored mitigation of oxidative stress and mitochondrial dysfunction. We treated old mice with NAC by oral gavage on alternate days for 4 weeks, followed by WNV infection via footpad, and analyzed WNV‐specific T cell response at day 7 and analyzed CD8^+^ T cells reactive to NS4b‐tet. As expected, fewer NS4‐tet^+^CD8^+^ T cells were found in dLN and blood of untreated old mice compared to adult mice (Figure 5a,b). The dLN of NAC‐treated old mice had larger numbers of NS4b^+^CD8^+^ T cells compared to untreated old mice (Figure 5b). Similarly, we noted a significantly higher number of circulating NS4b^+^CD8^+^ T cells in NAC‐treated old mice compared to untreated old mice (Figure 5b). Notably, the absolute numbers of NS4b^+^CD8^+^ T cells within the dLN and blood of NAC‐treated old mice were comparable to the levels observed in the adult mice (Figure 5b). The virus‐specific CD8^+^ T cells exert a robust cytotoxic function and require Granzyme B to clear the virus‐infected cells. We analyzed the intracellular expression of Granzyme B in NS4b‐tet^+^CD8^+^ T cells. We noted a nonsignificant trend of increased number of Granzyme B‐expressing NS4b‐tet^+^CD8^+^ T cells in the dLN in NAC‐treated old mice than untreated old mice (Figure 5c). NAC‐treatment also led to significantly higher numbers (> 2‐fold) of circulating Granzyme B + NS4b‐tet^+^CD8^+^ T cells in old mice compared to untreated old controls (Figure 5c). Further, circulating NS4b‐tet^+^CD8^+^ T cells from old mice had decreased expression of Granzyme B compared to their adult counterparts (Figure S10a,b), consistent with our original findings that old mice not only had fewer WNV‐specific T cells but also less cytotoxic capacity per cell (Brien et al. 2009). The NAC treatment restored Granzyme B expression to levels comparable to those found in adult NS4b‐tet^+^CD8^+^ T cells. Collectively, these data demonstrate that NAC‐treatment‐induced mitigation of oxidative stress successfully improved the WNV‐specific cytotoxic T cell response comparable to the level invoked in adult mice.

## 3 Discussion

In this study, we demonstrate that old LN stromal cells exhibit pronounced oxidative stress and mitochondrial dysfunction, which at least in part explain their inability to support Tn maintenance and function both in vitro and in vivo. Moreover, we show that the above defects are at least in part reversible, because antioxidant pretreatment of LN stromal cells improved Tn cell maintenance in vitro, and oral administration of NAC antioxidant treatment improved generation of effector T cells in vivo in response to WNV infection.

Complementary findings showed that old Tn cells were maintained and survived on par with adult Tn cells when co‐cultured with adult LN stromal cells in vitro. This provides another line of corroborating evidence to our prior in vivo results showing that old Tn cells harbor few demonstrable cell‐intrinsic defects (Jergovic et al. 2019) and that many of the defects that are read out as defects in adaptive immune responses lie with the disorganized old LN stromal cells (Becklund et al. 2016; Denton et al. 2022; Richner et al. 2015; Sonar et al. 2022; Thompson et al. 2019), the dysregulated old circulating mediators (Davies et al. 2018), and/or with the initial innate/inflammatory responses (Grolleau‐Julius et al. 2006; Jergovic et al. 2019; Li et al. 2012; Zhao et al. 2011).

One potentially interesting feature of LN stromal cell aging was that while these cells did not maintain CD8 Tn cell viability well, they did manage to maintain Tn phenotype similarly to the adult stromal cells, with the exception of CD62L, which was significantly lost from Tn cell surface in the presence of old, but not adult, stroma. Not much is known about the dependence of CD62L on Tn:stromal interactions, and this molecule is typically very prone to shedding under stress conditions, dominantly by the metalloproteinase ADAM17 (Chen et al. 1995; Kahn et al. 1994; Peschon et al. 1998). It will be of interest to determine whether this metalloproteinase may be overexpressed by old LN stromal cells.

While much has been learned about the embryonic development of lymph nodes and the developmental trajectories of stromal cells (De Martin et al. 2024; Onder and Ludewig 2018; Pikor et al. 2021), we know much less about the steady‐state maintenance of adult stromal subsets, or about their aging. One notable molecule involved in LN embryogenesis is LTβ, secreted by LT_i_ cells, and acting upon fibroblastic LT_o_ precursors to organize the LN anlage (Yoshida et al. 2002). Indeed, FRC‐restricted deletion of LTβR gene in Ccl19^Cre^LTβR^fl/fl^ mice resulted in structural disorganization and loss of T and B cells, which led to a subpar immune response to infection (Chai et al. 2013; Cremasco et al. 2014), indicating that changes in stromal cell function directly translate into defective T cell maintenance and function. In this study, we found reduced LTβ transcription in old LN. While agonistic LTβ signaling has been shown to fully substitute for the embryonic deletion of LT_i_ cells, we found that the same manipulation could partially improve Tn cell maintenance in vitro, but not their priming and differentiation into effector T cells. One interpretation of these results is that LN maintenance at an advanced age may be more complex and may require additional signaling via other pathways in order to improve the function of old LN stroma. Dissection of transcriptional changes and of cellular and molecular interactions leading to the loss of stromal function is in progress to address this issue.

It is pertinent to ask whether old LN stroma merely loses its supportive function for Tn homeostasis and function with aging, or whether it also may be actively adversely affecting Tn cells. Our initial experiments with tissue culture supernatant swaps did not decisively answer this question, and additional experimentation, outside the scope of this study, will be needed to dissect the impact of supernatants. Other experiments presented here, however, collectively argue in favor of the loss of supportive function. Specifically, antioxidant, mitochondrial function boosting, and LTβ agonistic signaling pretreatments of old stroma all significantly improved its Tn maintenance function. Moreover, IL‐7, even at low doses, was sufficient to singlehandedly improve Tn survival in co‐cultures with old stroma. These results are consistent with a loss of Tn supportive function of LN stroma with aging.

It is of interest that T cells, lymphocytes, and likely hematopoietic cells as a whole exhibit different aging patterns from mesenchymal cells that show stereotypical cellular senescence described originally for fibroblasts in culture (Hayflick and Moorhead 1961) and since defined as the senescence cell fate in vivo (Chaib et al. 2022; Wiley et al. 2016). It is therefore exceedingly important to rigorously establish both the manifestations of aging and the mechanisms of age‐related changes in a cell‐specific manner in ex vivo isolated as well as in vivo examined interacting cell types. Our discovery that stromal cell subsets, but not Tn cells, exhibit pronounced signs of oxidative stress and mitochondrial dysfunction is novel, particularly in the context of impaired interactions between these two cell types in homeostasis and in immune responses. Moreover, our work points out ways to ameliorate and correct the age‐related defects in lymph node stroma. These findings are both novel and physiologically relevant, as demonstrated by the improvement of lymph node stroma function in vitro and in vivo. A more detailed analysis of other markers of cellular senescence in these cell subsets is ongoing and should be helpful to inform additional cellular and molecular interventions to improve LN stromal function.

In conclusion, results presented in this study identify cellular stress mechanisms hampering the function of LN stromal cells and present successful initial attempts to restore that function. Consistent with previous work in the field (Rev. in Sonar et al. 2023), they further emphasize the need to understand and treat LN dysfunction with aging as an important contributor to reduced immune defenses and increased vulnerability to infection with aging.

## 4 Materials and Methods

### 4.1 Mice

Adult (2–4 months) and old (18–24 months) C57BL/6 mice were obtained from the National Institute of Aging breeding colony. All mice were housed under specific pathogen‐free conditions at the University Animal Care facility, University of Arizona, Tucson, AZ. All animal experiments were performed in compliance with the guidelines of the University of Arizona's Institutional Animal Care and Use Committee.

### 4.2 WNV Infection Model

Adult and old C57BL/6 mice were s.c. injected in both hind foot‐pads with WNV strain 385‐99 (1 × 10^3^ pfu) diluted in 50 μL USP saline. At day 7 postinfection, mice were euthanized with isoflurane, and blood, draining lymph nodes (popliteal and inguinal), and spleen were harvested.

### 4.3 Lymph Node Stromal Cell Isolation and Culture

For simultaneous analysis of antigen‐specific T cell response and stromal cells, one side of the LN was pushed through the 40 μm cell strainer, while those from the other side were digested with 0.2 mg/mL Liberase‐TL (Sigma, Saint Louis, MO) and 20 μg/mL DNase‐I (Sigma) in RPMI‐1640 plus 2% FBS (Omega Scientific, CA) at 37°C with continuous shaking for 60 min (Sonar et al. 2022). Tissue was dissociated with intermittent pipetting using a 1.0 mL pipette. Liberated cells were washed with RPMI‐1640 containing 10% FBS and passed through a 100 μm cell strainer. Cell viability was determined by trypan blue dye exclusion, and cell preparations consistently found more than 95% viable. Isolated single cell suspension was cultured in growth medium (minimum essential medium [MEM] containing 10% FBS) in a T‐25 tissue culture flask at 37°C and 5% CO_2_. The next day, the culture flask was washed thrice with growth medium to remove floating leukocytes and dying cells from the culture, leaving adherent stromal cells intact. The culture continued to be incubated for 5 days with replacement of growth medium every alternate day. At day 6, adherent cells (around 75%–80% confluence) were dislodged using 0.25% Trypsin, and a fraction of cells were stained with anti‐mouse CD45, anti‐mouse CD31, and anti‐mouse podoplanin to determine the purity of stromal cell populations (LEC, BEC, FRC, and DN cells). As depicted in Figure S1a, more than 99% of cells in the culture were CD45^−^ stromal cells, within which all four stromal cell populations were equivalently present between adult and old cell preparations. Cells from passages 3 to 7 were used for the experiments.

### 4.4 Purification of Naïve CD8 ^+^ T Cells

Peripheral lymph nodes and spleen were harvested from adult and old C57BL/6 mice and pushed through a 40 μm cell strainer using a syringe plunger. Single cells were washed, RBCs were lysed using ACK lysis buffer, washed with RPMI‐1640 containing 10% FBS, followed by a wash with MACS buffer (PBS with 0.2% BSA and 2 mM EDTA). Naïve CD8^+^ T cells were purified using a naïve CD8a^+^ T cell isolation kit (Miltenyi Biotec) according to the manufacturer's instructions. Purity of enriched cells was determined by flow cytometry analysis of CD8^+^CD62L^hi^CD44^lo^ cells, which were consistently over 97%–98%.

### 4.5 Lymph Node Stromal Cell‐T Cell Co‐Cultures

Purified primary LN stromal cells (passage 2–7) were cultured in a 96‐well flat‐bottom tissue culture plate (Corning) at a density of 2.5 × 10^3^ cells per well in MEM plus 10% FBS overnight at 37°C and 5% CO_2_. Wherever needed, cells were washed with growth medium and treated with NAC (1 mM), agonistic anti‐LTβR (2 μg/mL), mitoquinone (0.01, 0.1, and 1 μM), and urolithin‐A (1 and 10 μM) for 24 h. The next day, cells were washed thrice with PBS, followed by a final wash with growth medium and co‐cultured with 1 × 10^5^ purified naïve CD8^+^ T cells from adult and old C57BL/6 mice for 4 days, and the survival of CD8^+^ T cells in culture was analyzed by flow cytometry. Wherever indicated, purified IL‐7 was added to the culture to serve as a positive control for T cell survival in the culture. In some experiments, adult CD8^+^ Tn cells were co‐cultured with a monolayer prepared from a mix of different ratios of adult and old lymph node stromal cells. After day 4 of co‐culture, T cells were collected from the culture medium, washed, and treated with mouse Fc‐block for 30 min at room temperature (RT) followed by staining with antibodies directed to surface molecules. Cells were washed with FACS buffer followed by two washes with Annexin V‐binding buffer and incubated with APC conjugated Annexin V (Biolegend) and fixable cell viability Zombie Aqua (Invitrogen) dye for 15 min at RT. Cells were washed with Annexin V‐binding buffer and immediately acquired on LSR Fortessa (BD Bioscience). For intracellular BCL2, BAX, and Cleaved Caspase 3 staining, cells were fixed and permeabilized with Foxp3 fixation/permeabilization buffer set (eBioscience) as per the manufacturer's instructions.

### 4.6 Measurement of Mitochondrial Parameters

Peripheral LN (pooled inguinal, axillary and brachial LN) were harvested from adult (2–4 months), mid‐age (9–10 months), and old (18–21 months) C57BL/6 mice. The LN were digested with 0.2 mg/mL Liberase‐TL and 20 μg/mL DNase‐I in RPMI‐1640 plus 2% FBS at 37°C with continuous shaking for 60 min. The single cell suspension was washed with RPMI‐1640 containing 10% FBS. Cells were counted and about 1 × 10^6^ cells were used for staining with different cellular and mitochondrial probes at 37°C in 5% CO_2_ incubator. Cells were treated with 1 μM 2′,7′‐dichlorofluorescin‐diacetate (DCF‐DA; Sigma) for 15 min for cellular ROS detection; with 5 μM MitoSOX‐Red (Invitrogen) for 30 min for mitochondrial ROS; with 200 nM MitoTracker Green‐FM (Invitrogen) for 30 min for mitochondrial mass; and with 200 nM MitoTracker Deep Red‐FM (Invitrogen) for 30 min for mitochondrial membrane potential. After the treatment of the indicated probes, cells were washed and stained for cell surface markers to identify stromal and immune cell populations and analyzed by flow cytometry. Mitochondrial membrane potential was calculated by a ratio of mitochondrial mass derived from mitochondrial membrane potential‐dependent (MitoTracker Deep Red‐FM) and ‐independent (MitoTracker Green‐FM) dyes (Yu et al. 2020).

### 4.7 NAC Treatment in Old Mice

Old (18–20 months) C57BL/6 male mice were given NAC at 150 mg/kg through oral gavage every alternate day for four continuous weeks (Cao et al. 2012). Control old mice were given drinking water through oral gavage at the same times. After 4 weeks, all mice were s.c. injected in both hind foot‐pads with WNV strain 385‐99 (1 × 10^3^ pfu) diluted in 50 μL USP saline. At day 7 postinfection, mice were euthanized with isoflurane, and blood and draining lymph nodes (popliteal and inguinal) were harvested, and primary T cell and stromal cell responses were analyzed by flow cytometry.

### 4.8 Agonistic Anti‐LTβR Treatment in Old Mice

Old (18–20 months) C57BL/6 male mice were i.p. injected with agonistic anti‐LTβR antibody (100 μg/mice) at days 9, 6, and 3 days before challenged in both hind foot‐pad with WNV strain 385‐99 (1 × 10^3^ pfu) diluted in 50 μL USP saline. At day 7 postinfection, mice were euthanized with isoflurane and blood and draining lymph nodes (popliteal and inguinal) were harvested, and primary T cell and stromal cell responses were analyzed by flow cytometry.

### 4.9 Determination of Apoptosis in Ex Vivo Isolated T Cells

Peripheral LN (pool of axillary, inguinal, and brachial LN) were harvested from euthanized adult (3–4 months) and old (19–21 months) male C57BL/6 mice. LN were passed through a 40 μm cell strainer using a syringe plunger and washed. About 1 × 10^6^ cells were stained with antibodies directed to surface antigen followed by washing with Annexin V binding buffer. Cells were stained with Annexin V‐APC and fixable live dead‐Zombie Aqua dye in Annexin V binding buffer. Cells were washed with Annexin V binding buffer and acquired immediately on a flow cytometer. Staining for CD62L and CD44 on the surface of CD3^+^CD4^+^ and CD3^+^CD8^+^ T cells was used to define naïve (CD62L^hi^CD44^lo^), central memory (CD62L^hi^CD44^hi^), and effector memory (CD62^lo^CD44^hi^) T cell populations. For intracellular cleaved caspase 3 staining, cells were fixed and permeabilized with Foxp3 fixation/permeabilization buffer set (eBioscience) as per the manufacturer's instructions.

### 4.10 Flow Cytometry

Single cell suspensions were prepared from peripheral or draining lymph nodes and spleen by passing tissue through a 40 μm cell strainer using a syringe plunger. Blood and spleen cells were treated with ACK lysis buffer to lyse RBCs. About 1–2 × 10^6^ cells were stained with mouse anti‐CD16/32 Fc block for 20 min at 4°C, followed by staining with fluorochrome‐conjugated antibodies directed at surface antigens. Cells were washed with FACS buffer (PBS +2% FBS) and PBS, stained with 1:1000 dilutions of fixable live dead stains (BioLegend). For intracellular staining, cells were fixed and permeabilized with Foxp3 fixation/permeabilization buffer set (eBioscience) as per the manufacturer's instructions. Intracellular staining was performed in Foxp3 permeabilization buffer. Cells were washed twice with Foxp3 permeabilization buffer, followed by a wash with FACS buffer. Cells were resuspended in FACS buffer containing 20 μL CountBright absolute counting beads (Invitrogen). Fluorescence minus one (FMO) controls were run to position the gates in the flow cytometry plots. Samples were acquired either on LSR Fortessa (BD Bioscience) or Cytek Aurora spectral cytometer (Cytek Bioscience). Data were analyzed using FlowJo v10.8.0 (TreeStar). The absolute number of cells was determined as: Cells/μL = [((cell count/counting bead count) × (counting bead volume/cell volume)) × counting bead concentration (beads/μL)].

### 4.11 Statistics

Data are expressed as mean ± standard error of mean (SEM). As indicated in the figure legends, statistical analysis was performed by one‐ or two‐way analysis of variance (ANOVA) followed by Tukey's multiple comparison correction test or Mann–Whitney *U* test using GraphPad Prism 10 software. Group comparisons with *p* < 0.05 were considered statistically significant.

Detailed methods, the list and source of antibodies and reagents can be found in the Supporting Information and Table S1.

## Author Contributions

S.A.S. and J.Ž.N. designed research; S.A.S., R.B., H.L.T., C.P.C., J.L.U., C.P.C., and M.J. performed research; S.A.S., H.L.T., and J.Ž.N. analyzed data; S.A.S. and J.Ž.N. wrote paper; all authors edited paper; J.Ž.N. conceived project, supervised the study, and acquired funding.

## Conflicts of Interest

The authors declare no conflicts of interest.

## Supporting information


Data S1.

## Data Availability

All data are included in the manuscript and Supporting Information.
