# Supplementary material for: Age‐Related Oxidative Stress and Mitochondrial Dysfunction in Lymph Node Stromal Cells Limit the Peripheral T Cell Homeostatic Maintenance and Function
Source: Aging Cell. 2025 May 21;24(8):e70100. doi: 10.1111/acel.70100 (PMC12341786; doi:10.1111/acel.70100)
Supplement: Supplementary file 1 — Data S1. [file ACEL-24-e70100-s001.pdf]

Supplementary Information for

Age-related oxidative stress and mitochondrial dysfunction in lymph node stromal cells limit the peripheral T cell homeostatic maintenance and function

Sandip Ashok Sonar, Ruchika Bhat, Heather L. Thompson, Christopher P. Coplen, Jennifer L. Uhrlaub, Mladen Jergovic, and Janko Ž. Nikolich.

Correspondence to : Janko Ž. Nikolich

Email: nikolich@arizona.edu

This PDF file includes:

Materials and Methods

Figures S1 to S10

Table S1

Materials and Methods.

Antibodies and Reagents. Following fluorochrome dye or biotin-labeled anti-mouse antibodies were used in the study: CD3 (17A2), CD4 (GK1.5), CD8 (53-6.7), CD62L (MEL-14), CD44 (IM7), CD25 (PC61), CD69 (H1.2F3), CD127 (A7R34), CCR7 (4B12), CD45 (30-F11), Ter-119 (TER-119), CD31 (390), Podoplanin (8.1.1), Granzyme B (QA16A02), and APC-conjugated Annexin V were purchased from BioLegend. CD5 (53-7.3) was from BD Biosciences, BCL2 (10C4) was from eBioscience, and cleaved caspase 3 (269518) was from R&D Systems. PE conjugated H-2D(b)-NS4b$_{2488}$-tetramer was obtained from Emory NIH tetramer facility. Agonistic anti-LTβR (AF-H6) was purchased from Biogen (ref no. 4749-70) (Kumar et al., 2015). Zombie Aqua fixable viability dye (Biolegend) was used to separate live and dead cells. The detailed list and source of antibodies and reagents are provided in Table S1.

Analysis of survival of Tn cells in vitro in the presence of lymph node stromal cell supernatant. $CD4^+$ and $CD8^+$ Tn ($CD62L^{hi}CD44^{lo}$) cells from the spleen and lymph nodes of adult (2-3 mo) and old (20-21 mo) mice were enriched using Pan T cell enrichment kit (Miltenyi Biotec). 1 x 10^5 Tn cells were cultured in a 96-well U-bottom plate in RPMI-1640 containing 10% FBS, 2 mM L-glutamine, and 1 mM sodium pyruvate for 48 hours. The titrated (1:200 dilutions) amount of culture supernatant (filtered twice through a 0.2µm filter) derived from the adult or old primary lymph node stromal cell culture at passage 3-5 was added to the culture. After 48 hours, Tn cells were harvested and stained with Fc-block for 30 minutes at room temperature (RT) followed by surface molecule staining. Cells were washed with FACS buffer followed by two washes with Annexin V-binding buffer and incubated with APC-conjugated Annexin V (Biolegend) and fixable cell viability Zombie Aqua (Invitrogen) dye for 15 minutes at RT. Cells were washed with Annexin V-binding buffer and immediately acquired on LSR Fortessa (BD Bioscience).

qRT-PCR mini-array for mRNA expression analysis. Inguinal, axillary, and popliteal lymph nodes were harvested from adult (3 mo) and old (19 mo) C57BL/6 mice, homogenized, and RNA was isolated using RNeasy Mini Kit (Qiagen). The quantity and quality of RNA samples were determined by nanodrop and RNA Bio analyzer. 250 ng RNA from each group was used for RT2 PCR array assay per manufacturer's instructions. Briefly, genomic DNA was removed and subsequently subjected for cDNA synthesis using RT2 First strand kit reagents (Qiagen). Quantitative PCR amplification of cDNA samples was performed using Qiagen's RT2 PCR array in 96-well plate format and RT2 SYBR Green master mix (Qiagen). Cycling conditions were a cycle at 95°C for 10 minutes for the activation of Hot Start Taq DNA polymerase followed by 40

cycles of denaturation (95°C for 15 seconds) and annealing and extension (60°C for 1 minute). Data were exported and analyzed using Qiagen’s GeneGlobe Data Analysis Center.

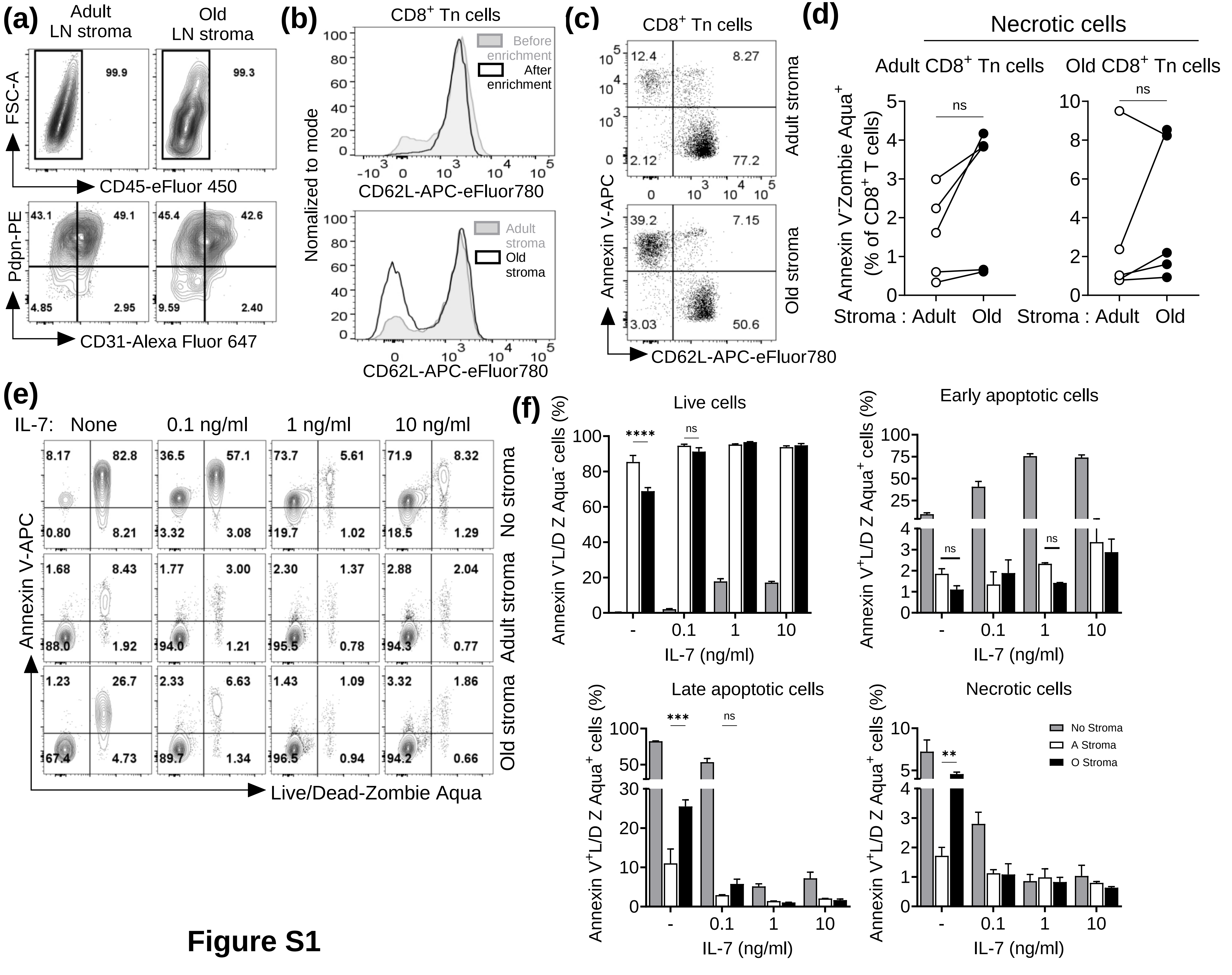

(a)
Adult LN stroma
Old LN stroma
FSC-A
CD45-eFluor 450
Pdpn-PE
CD31-Alexa Fluor 647
(b)
CD8+ Tn cells
Before enrichment
After enrichment
Adult stroma
Old stroma
Nomalized to mode
CD62L-APC-eFluor780
(c)
CD8+ Tn cells
Adult stroma
Old stroma
Annexin V-APC
CD62L-APC-eFluor780
(d)
Necrotic cells
Adult CD8+ Tn cells
Old CD8+ Tn cells
Annexin V-Zombie Aqua+ (% of CD8+ T cells)
ns
Stroma : Adult Old
(e)
IL-7: None 0.1 ng/ml 1 ng/ml 10 ng/ml
No stroma
Adult stroma
Old stroma
Annexin V-APC
Live/Dead-Zombie Aqua
(f)
Live cells
Early apoptotic cells
Late apoptotic cells
Necrotic cells
Annexin V-L/D Z Aqua- cells (%)
Annexin V+L/D Z Aqua+ cells (%)
IL-7 (ng/ml)
No Stroma
A Stroma
O Stroma

Figure S1

Figure S1. Aging lymph node stromal cells limits peripheral T cell survival. (a) Representative flow cytometry plots (gated on live cells) show the purity of adult and old stromal cells in culture. More than 99% cells in the culture were CD45- stromal cells (top row). Bottom row depicts staining of CD31 and Pdpn within CD45- stromal cells. Both adult and old stromal cell cultures represent similar fractions of major stromal cell populations; FRC ($Pdpn^+CD31^-$), LEC ($Pdpn^+CD31^+$), BEC ($Pdpn^-CD31^+$), and DN ($Pdpn^-CD31^-$). **(b)** Representative histogram shows CD62L expression on the surface of adult $CD8^+$ T cells before and after the enrichment (top), and after 4 days of co-culture with adult or old stromal cells (bottom). **(c)** Representative dot plot shows staining of Annexin-V and CD62L on CD8+ Tn cells after 4 days of co-culture. **(d)** Frequency of necrotic Tn cells (Annexin V-Live/Dead-Zombie Aqua+) after 4 days of co-culture with adult and old Ln stromal cells was shown. **(e)** Representative flow cytometry plots (gated on live, lymphocytes, $CD8^+$ T cells) show staining of Annexin V and Live/Dead-Zombie Aqua indicating live (Annexin V^-Live/$Dead^-$), early apoptotic (Annexin V^+Live/$Dead^-$), late apoptotic (Annexin V^+Live/$Dead^+$), and necrotic (Annexin V^-Live/$Dead^+$) $CD8^+$ T cells co-cultured with adult and old LN stromal cells with or without exogenous IL-7 in an indicated concentration for 4 days. **(f)** Percentages of $CD8^+$ T cells with live (Annexin V^-Live/$Dead^-$), early apoptotic (Annexin V^+Live/$Dead^-$), late apoptotic (Annexin V^+Live/$Dead^+$), and necrotic phenotype (Annexin V^-Live/$Dead^+$) were plotted. Numbers next to the gate or in the quadrant indicate the percentage of the corresponding cell population with the gate (a, c, e). Data is representative of 3-4 independent experiments. Man Whitney U test (d), Two-way ANOVA followed by Tukey's multiple comparison correction test (f). ns- non-significant, * $p \leq 0.05$, ** $p \leq 0.01$, *** $p \leq 0.001$, and **** $p \leq 0.0001$.

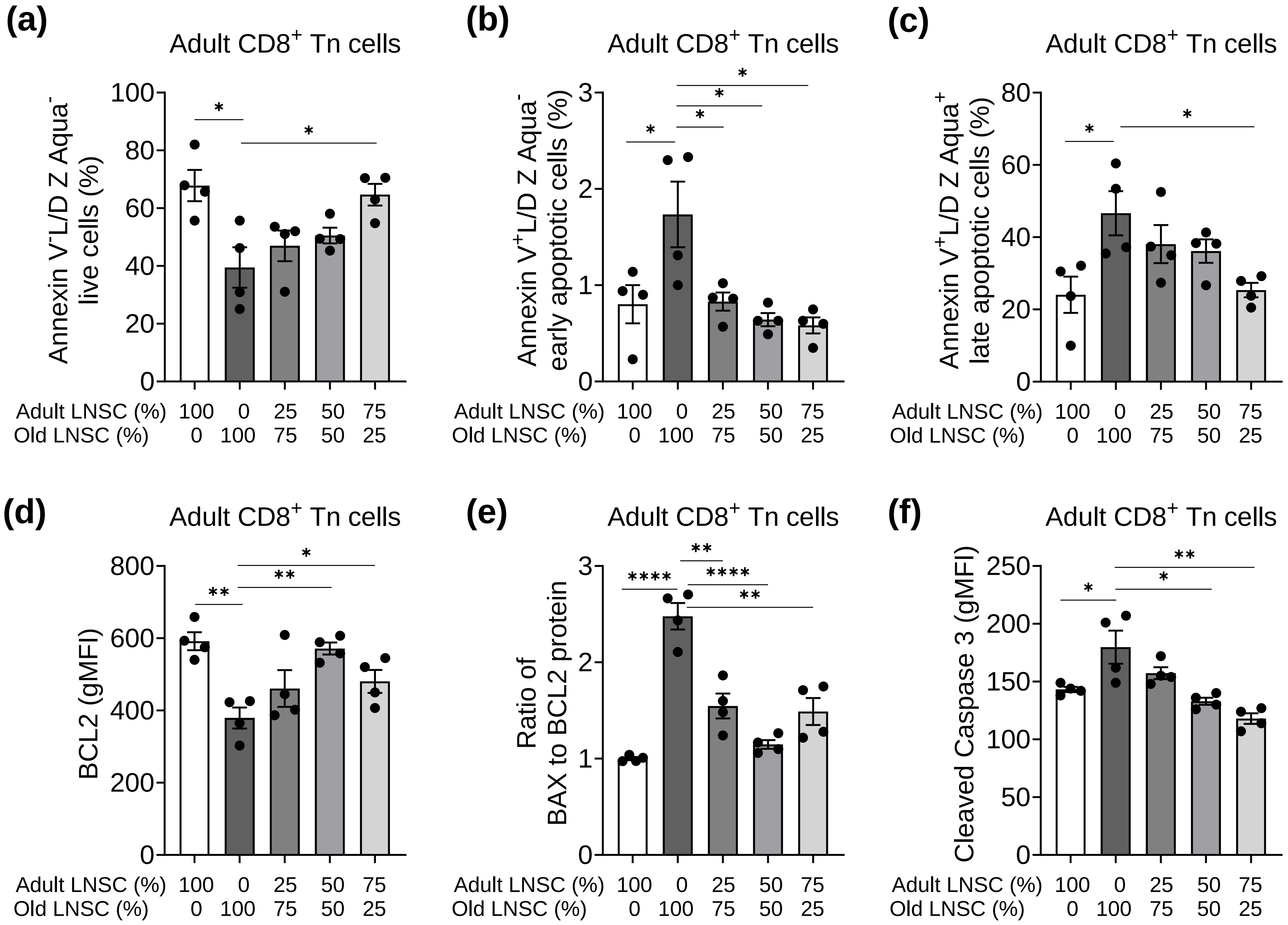
(a)
Adult CD8+ Tn cells
Annexin V-L/D Z Aqua- live cells (%)
(b)
Adult CD8+ Tn cells
Annexin V+L/D Z Aqua- early apoptotic cells (%)
(c)
Adult CD8+ Tn cells
Annexin V+L/D Z Aqua+ late apoptotic cells (%)
(d)
Adult CD8+ Tn cells
BCL2 (gMFI)
(e)
Adult CD8+ Tn cells
Ratio of BAX to BCL2 protein
(f)
Adult CD8+ Tn cells
Cleaved Caspase 3 (gMFI)
Adult LNSC (%) 100 0 25 50 75
Old LNSC (%) 0 100 75 50 25

Figure S2

Figure S2. Improvement in the survival of $CD8^+$ Tn cells in the co-culture of mixed adult and old lymph node stromal cells. Purified adult $CD8^+$ Tn cells were co-cultured with a monolayer prepared from adult, old, or different ratios of adult and old lymph node stromal cells for 4 days, and survival of Tn cells was analyzed by flow cytometry. Data show the percentage of Annexin-V^-Live/$Dead^-$ live **(a)**, Annexin-V^+Live/$Dead^-$ early apoptotic **(b)**, Annexin-V^+Live/$Dead^+$ late apoptotic **(c)** $CD8^+$ Tn cells. Data show the geometric mean fluorescence intensity (gMFI) of intracellular BCL2 **(d)**, a ratio of BAX to BCL2 protein **(e)**, and gMFI of cleaved caspase 3 **(f)** in adult $CD8^+$ Tn cells. Data is representative of three independent experiments. Unpaired t test (a-f). * $p \leq 0.05$, ** $p \leq 0.01$, *** $p \leq 0.001$, and **** $p \leq 0.0001$.

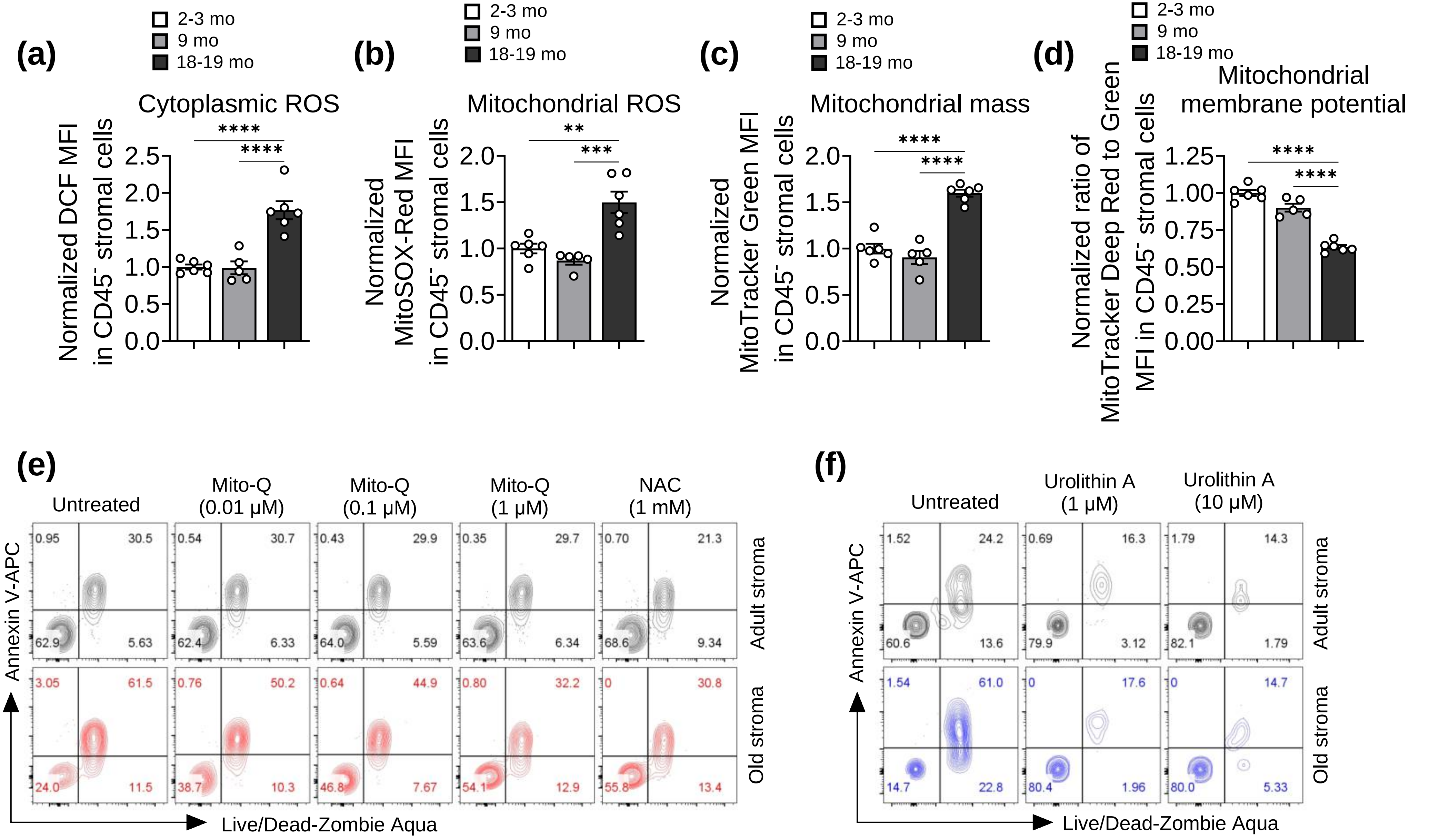
(a)
2-3 mo
9 mo
18-19 mo
Cytoplasmic ROS
Normalized DCF MFI in CD45⁻ stromal cells
(b)
Mitochondrial ROS
Normalized MitoSOX-Red MFI in CD45⁻ stromal cells
(c)
Mitochondrial mass
Normalized MitoTracker Green MFI in CD45⁻ stromal cells
(d)
Mitochondrial membrane potential
Normalized ratio of MitoTracker Deep Red to Green MFI in CD45⁻ stromal cells
(e)
Untreated
Mito-Q (0.01 μM)
Mito-Q (0.1 μM)
Mito-Q (1 μM)
NAC (1 mM)
Adult stroma
Old stroma
Annexin V-APC
Live/Dead-Zombie Aqua
(f)
Untreated
Urolithin A (1 μM)
Urolithin A (10 μM)
Adult stroma
Old stroma
Annexin V-APC
Live/Dead-Zombie Aqua

Figure S3

Figure S3. Modulation of mitochondrial function by mitoquinone and Urolithin-A in lymph node stromal cells improves their ability to support Tn cell survival in co-culture. **(a, b)** Peripheral LN (pooled inguinal, axillary and brachial LN) were digested with Liberase-TL and DNase-I and single cells suspension were treated with **(a)** 2',7'-dichlorofluorescin-diacetate (DCF-DA; 1 μM) for 15 minutes, **(b)** MitoSOX-Red (5 μM) for 30 minutes, **(c, d)** MitoTracker Green-FM (200 nM) and MitoTracker Deep Red-FM (200 nM) for 30 minutes at 37'C in 5% CO2 incubator. Cells were washed and stained for surface markers to identify stromal cell populations and analyzed by flow cytometry. Data show level of **(a)** cellular (cytoplasmic) ROS, **(b)** mitochondrial ROS, **(c)** mitochondrial mass, and **(d)** mitochondrial membrane potential in CD45- stromal cells from young adult (2-3 mo), mid-age (9 mo), and old (18-19 mo) mice. Data represent geometric mean fluorescence intensity of indicated molecules normalized to 2-3 mo group. Each dot represents an individual mouse. **(e, f)** Adult and old lymph node stromal cells were treated with the indicated concentrations of **(e)** Mitoquinone (Mito-Q) or N-Acetyl Cysteine (NAC; 1 mM), and **(f)** Urolithin-A (Uro-A) for 24 hours, cultures were washed, and purified adult $CD8^+$ Tn cells were co-cultured with treated and untreated stromal cells for 4 days. **(e, f)** Representative flow cytometry plots show staining of Annexin-V and Live/Dead-Zombie Aqua in $CD8^+$ Tn cells. Numbers in the quadrants indicate the percentage of the indicated cell population (e, f). Data are representative of 3-4 independent experiments and expressed as mean ± s.e.m. ANOVA followed by Tukey's multiple comparison correction test. ns- non-significant, * $p \leq 0.05$, ** $p \leq 0.01$, *** $p \leq 0.001$, and **** $p \leq 0.0001$ (a-d).

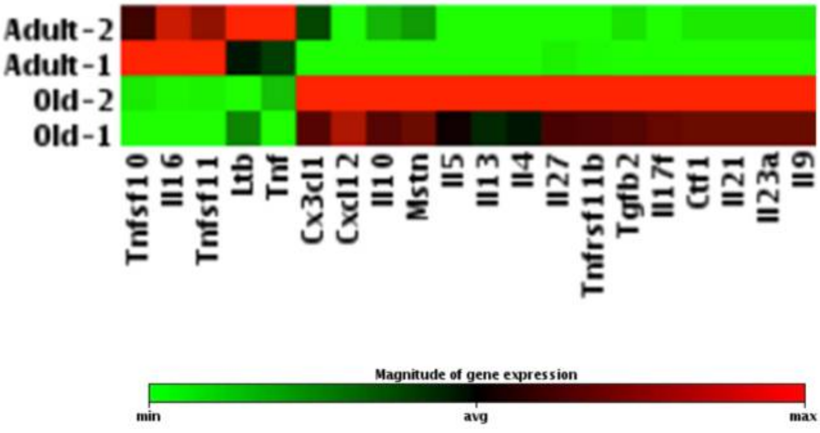
Adult-2
Adult-1
Old-2
Old-1
Tnfsf10
Il16
Tnfsf11
Ltb
Tnf
Cx3cl1
Cxcl12
Il10
Mstn
Il5
Il13
Il4
Il27
Tnfrsf11b
Tgfb2
Il17f
Ctf1
Il21
Il23a
Il9
Magnitude of gene expression
min
avg
max

Figure S4

Figure S4. Gene expression analysis in whole lymph nodes. RNA was extracted from the pooled inguinal, axillary, and popliteal lymph nodes from adult (3 mo) and old (19 mo) C57BL/6 mice, and mRNA expression was analyzed using Qiagen's RT2-PCR array. A heat map show the expression of indicated genes in adult and old lymph node samples. The old lymph node exhibit downregulation of Tnsfsf10, Il16, Tnfsf11, Ltb, and Tnf, and upregulated transcripts for Cx3cl1, Cxcl12, interleukins (Il4, Il5, Il9, Il10, Il13, Il17f, Il21, and Il23a), Mstn, Tnfsf11b, Tgfb2, Ctf1 relative to adult lymph node. The data represent one experiment with n=2 mice per group.

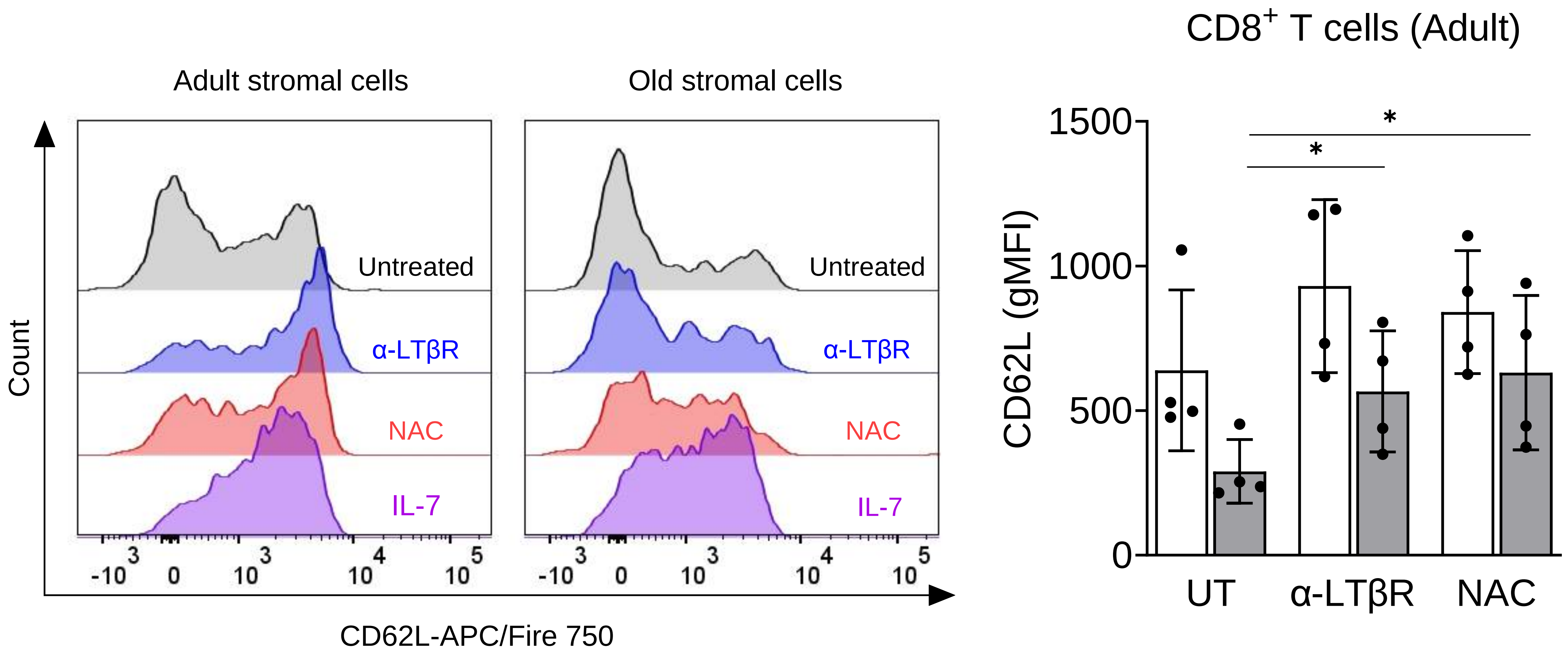

Adult stromal cells
Old stromal cells
Untreated
α-LTβR
NAC
IL-7
Count
-10^3
0
10^3
10^4
10^5
CD62L-APC/Fire 750
CD8+ T cells (Adult)
CD62L (gMFI)
1500
1000
500
0
*
*
UT
α-LTβR
NAC

Figure S5

Figure S5. Old LN stromal cells affect the surface expression of CD62L on co-cultured adult naïve $CD8^+$ T cells. Representative flow cytometry histograms denote CD62L expression on the surface of naïve $CD8^+$ T cells co-cultured for 4 days with adult and old LN stromal cells (left). Data show geometric MFI of CD62L-APC/Fire 750 on the surface of $CD8^+$ T cells co-cultured for 4 days with adult (open bars) and old (filled bars) LN stromal cells. Data pooled from 4 independent experiments and expressed as mean ± s.e.m. Each dot represents the mean of duplicate measurements performed in each experiment. Two-way ANOVA followed by Tukey's multiple comparison correction test. ns- non-significant, * $p \leq 0.05$, ** $p \leq 0.01$, *** $p \leq 0.001$, and **** $p \leq 0.0001$.

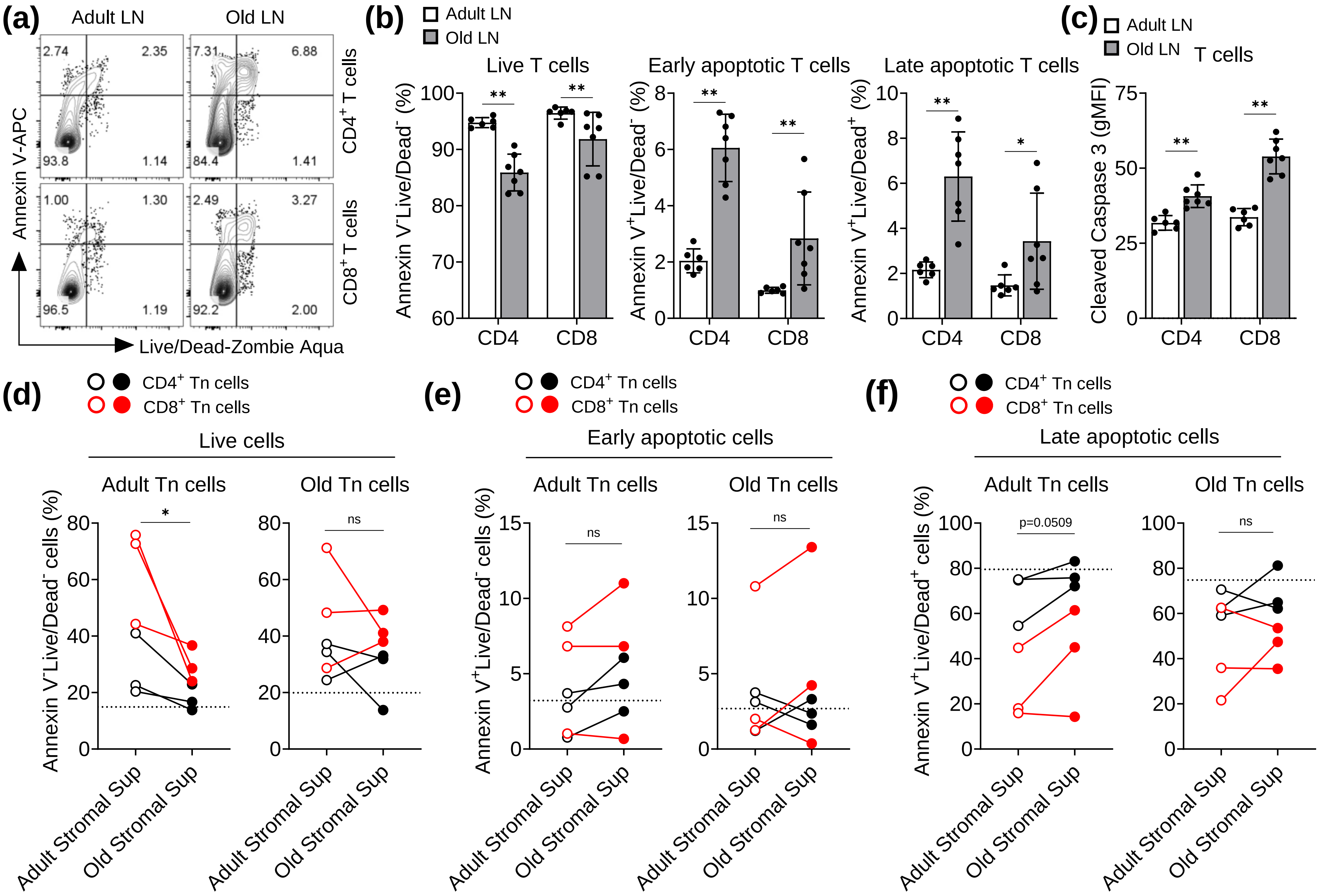
(a)
Adult LN
Old LN
2.74
2.35
93.8
1.14
7.31
6.88
84.4
1.41
CD4+ T cells
1.00
1.30
96.5
1.19
2.49
3.27
92.2
2.00
CD8+ T cells
Annexin V-APC
Live/Dead-Zombie Aqua
(b)
Adult LN
Old LN
Live T cells
Early apoptotic T cells
Late apoptotic T cells
Annexin V-Live/Dead- (%)
Annexin V+Live/Dead- (%)
Annexin V+Live/Dead+ (%)
CD4
CD8
(c)
T cells
Cleaved Caspase 3 (gMFI)
(d)
CD4+ Tn cells
CD8+ Tn cells
Live cells
Adult Tn cells
Old Tn cells
Annexin V-Live/Dead- cells (%)
Adult Stromal Sup
Old Stromal Sup
ns
(e)
Early apoptotic cells
Annexin V+Live/Dead- cells (%)
(f)
Late apoptotic cells
Annexin V+Live/Dead+ cells (%)
p=0.0509

Figure S6

Figure S6. Naïve CD4+ and CD8+ T cells from the old LN exhibit increased vulnerability to apoptosis. (a) Representative flow cytometry plots (gated on live, lymphocytes, $CD3^+$, $CD8^+$ or CD4+ cells) show staining of Annexin V and Live/Dead-Zombie Aqua in $CD4^+$ (top row) and $CD8^+$ (bottom row) T cells from adult and old pLN. Numbers in the quadrant indicate the percentage of the corresponding cell population within the gate. **(b)** The percentage of live (Annexin V^-Live/$Dead^-$), early apoptotic (Annexin V^+Live/$Dead^-$), and late apoptotic (Annexin V^+Live/$Dead^+$) $CD4^+$ and $CD8^+$ T cells within adult (open histogram) and old (filled histogram) LN were shown. **(c)** Levels of intracellular cleaved caspase 3 within $CD4^+$ and $CD8^+$ T cells from pLN of adult (open histogram) and old (filled histogram) mice were shown. Data is representative of 2 independent experiments and expressed as mean ± s.e.m. Each dot represents an individual mouse. **(d-f)** Purified adult $CD4^+$ and $CD8^+$ Tn ($CD62L^{hi}CD44^{lo}$) cells were cultured with culture supernatant (sup) of adult or old lymph node stromal cells for 48 hours, and the survival of Tn cells was analyzed by flow cytometry. The percentage of Annexin V^-Live/$Dead^-$ live **(d)**, Annexin V^+Live/$Dead^-$ early apoptotic **(e)**, and Annexin V^+Live/$Dead^+$ late apoptotic **(f)** $CD4^+$ (black circles) and $CD8^+$ (red circles) Tn cells were shown. Dotted horizontal line denotes the percentages of cells cultured in the absence of stromal cell supernatant (d-f). Data represents 3 independent experiments (d-f). Man-Whitney U test (b), paired t-test (d-f). ns-non-significant, * p ≤0.05, ** p ≤0.01, *** p ≤0.001, and **** p ≤0.0001.

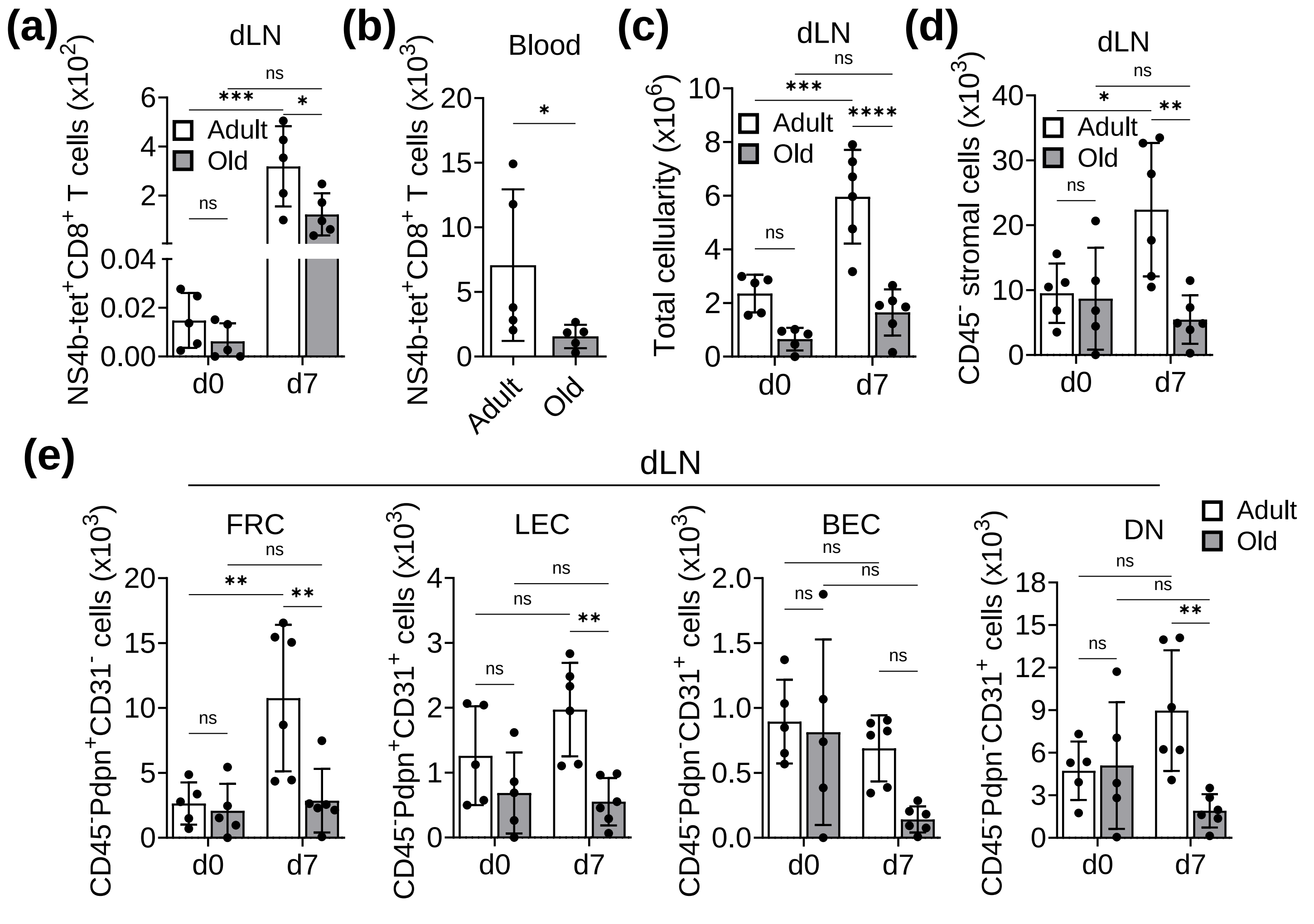
(a)
dLN
NS4b-tet+CD8+ T cells (x10²)
Adult
Old
d0
d7
(b)
Blood
NS4b-tet+CD8+ T cells (x10³)
Adult
Old
(c)
dLN
Total cellularity (x10⁶)
(d)
dLN
CD45- stromal cells (x10³)
(e)
dLN
FRC
LEC
BEC
DN
CD45-Pdpn+CD31- cells (x10³)
CD45-Pdpn+CD31+ cells (x10³)
CD45-Pdpn-CD31+ cells (x10³)
CD45-Pdpn-CD31+ cells (x10³)

Figure S7

Figure S7. Old lymph node stromal cells respond poorly to West Nile virus infection. Adult (2-4 mo) and old (18-20 mo) C57BL/6 mice were infected with WNV via s.c. in hind foot-pads and T cell response in blood and dLN were analyzed at 7 d.p.i. **(a, b)** Absolute numbers of H-2D(b)-NS4b-tetramer$^+$CD8$^+$ T cells in **(a)** dLN at 0 and 7 d.p.i. and **(b)** blood at 7 d.p.i. were shown. **(c)** total live cells, **(d)** CD45$^-$ stromal cells, and **(e)** FRC (CD45$^-$Pdpn$^+$CD31$^-$), LEC (CD45$^-$Pdpn$^+$CD31$^+$), BEC (CD45$^-$Pdpn$^-$CD31$^+$), and DN (CD45$^-$Pdpn$^-$CD31$^-$) cells from dLN were calculated and plotted. Data is representative of 3 independent experiments and expressed as mean ± s.e.m. Each dot represents an individual mouse. Two-way ANOVA followed by Tukey's multiple comparison correction test (a-d), Man Whitney U test (e). ns- non-significant, * p ≤0.05, ** p ≤0.01, *** p ≤0.001, and **** p ≤0.0001.

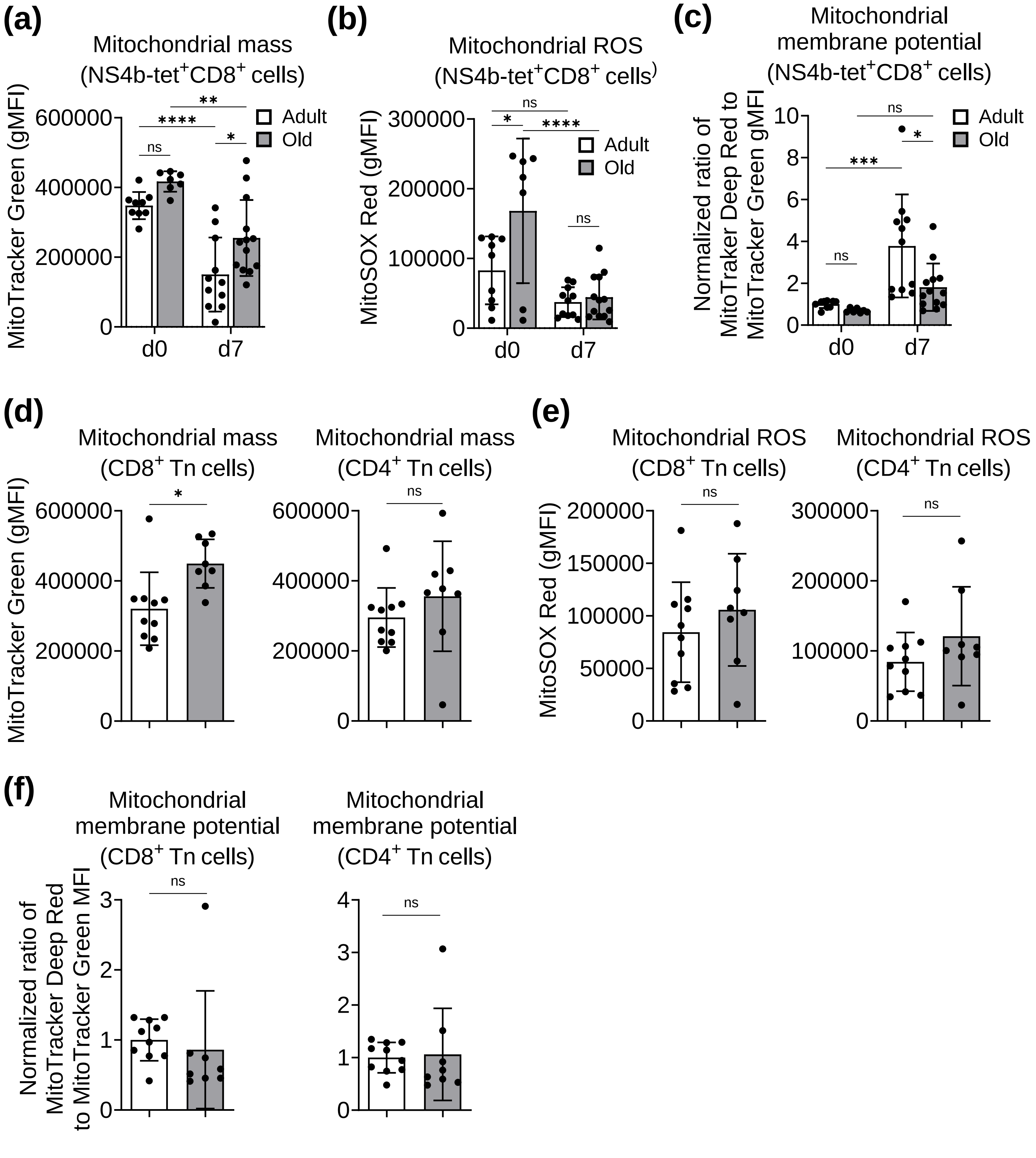

(a)
Mitochondrial mass
(NS4b-tet+CD8+ cells)
MitoTracker Green (gMFI)
Adult
Old
d0
d7
(b)
Mitochondrial ROS
(NS4b-tet+CD8+ cells)
MitoSOX Red (gMFI)
(c)
Mitochondrial
membrane potential
(NS4b-tet+CD8+ cells)
Normalized ratio of
MitoTraker Deep Red to
MitoTracker Green gMFI
(d)
Mitochondrial mass
(CD8+ Tn cells)
Mitochondrial mass
(CD4+ Tn cells)
(e)
Mitochondrial ROS
(CD8+ Tn cells)
Mitochondrial ROS
(CD4+ Tn cells)
(f)
Mitochondrial
membrane potential
(CD8+ Tn cells)
Mitochondrial
membrane potential
(CD4+ Tn cells)
Normalized ratio of
MitoTracker Deep Red
to MitoTracker Green MFI

Figure S8

Figure S8. T cells do not exhibit defects in mitochondrial function. Adult (2-4 mo) and old (18-20 mo) C57BL/6 mice were infected with WNV via s.c. in hind foot-pads, and T cell response in dLN was analyzed at 7 d.p.i. **(a)** Mitochondrial mass, **(b)** mitochondrial ROS, and **(c)** mitochondrial membrane potential were analyzed in H-2D(b)-NS4b-tetramer^{+}CD8^{+} T cells in dLN at 0 and 7 d.p.i.. **(d)** Mitochondrial mass, **(e)** mitochondrial ROS, and **(f)** mitochondrial membrane potential were analyzed in CD62L^{hi}CD44^{lo} naïve CD8^{+} (left) or CD4^{+} (right) T cells from adult and old LN. Data are pooled results from 2 independent experiments and expressed as mean ± s.e.m. Each dot represents an individual mouse. Two-way ANOVA followed by Tukey's multiple comparison correction test (a-c), Man Whitney U test (d-f). ns- non-significant, * p ≤0.05, ** p ≤0.01, *** p ≤0.001, and **** p ≤0.0001.

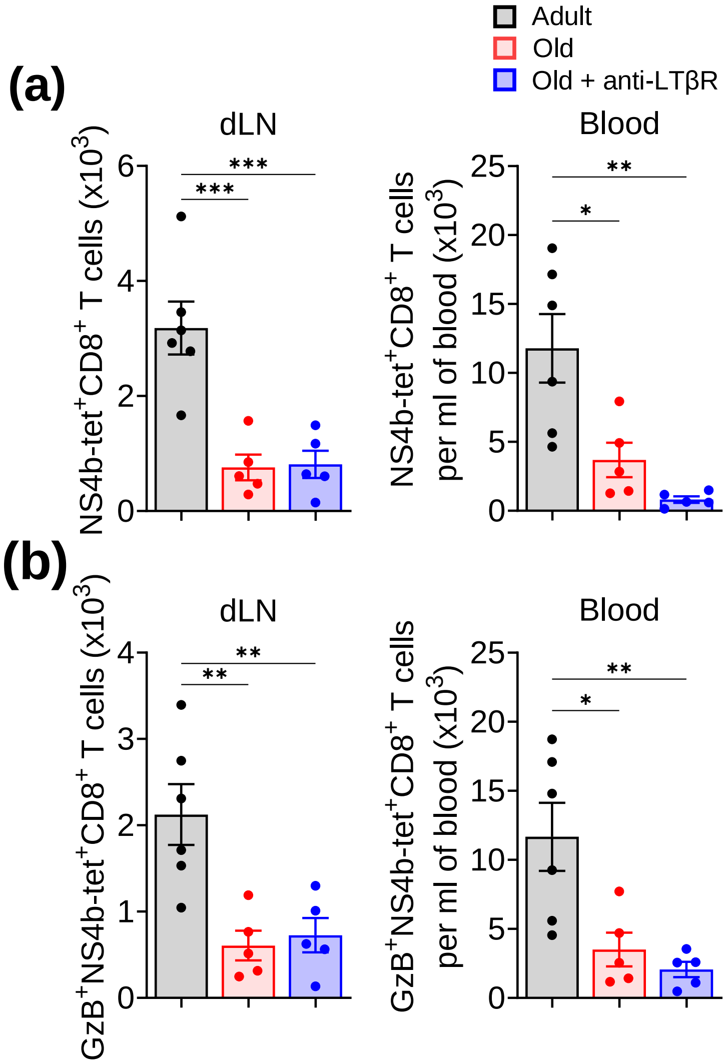
Adult
Old
Old + anti-LTβR
(a)
dLN
Blood
NS4b-tet+CD8+ T cells (x10³)
NS4b-tet+CD8+ T cells per ml of blood (x10³)
***
***
**
*
(b)
dLN
Blood
GzB+NS4b-tet+CD8+ T cells (x10³)
GzB+NS4b-tet+CD8+ T cells per ml of blood (x10³)
**
**
**
*

Figure S9

Figure S9. Agonistic anti-LTβR treatment in old mice failed to boost West Nile virus-specific T cells. Old (18-20 mo) C57BL/6 male mice were i.p. injected with agonistic anti-LTβR antibody (100 μg/mice) at days 9, 6, and 3 before being challenged with 1000 pfu of West Nile virus in both hind foot-pads. At 7 d.p.i., WNV-specific T cell response was analyzed. **(a)** Absolute numbers of H-2D(b)-NS4b-tetramer$^+$CD8$^+$ T cells in the dLN (left) and blood (right) were shown. **(b)** Absolute numbers of granzyme B$^+$H-2D(b)-NS4b-tetramer$^+$CD8+ T cells in the dLN (left) and blood (right) were shown. Each dot represents individual mice. One-way ANOVA followed by Tukey's multiple comparison test (A, B). ns- non-significant, * $p \leq 0.05$, ** $p \leq 0.01$, *** $p \leq 0.001$, and **** $p \leq 0.0001$.

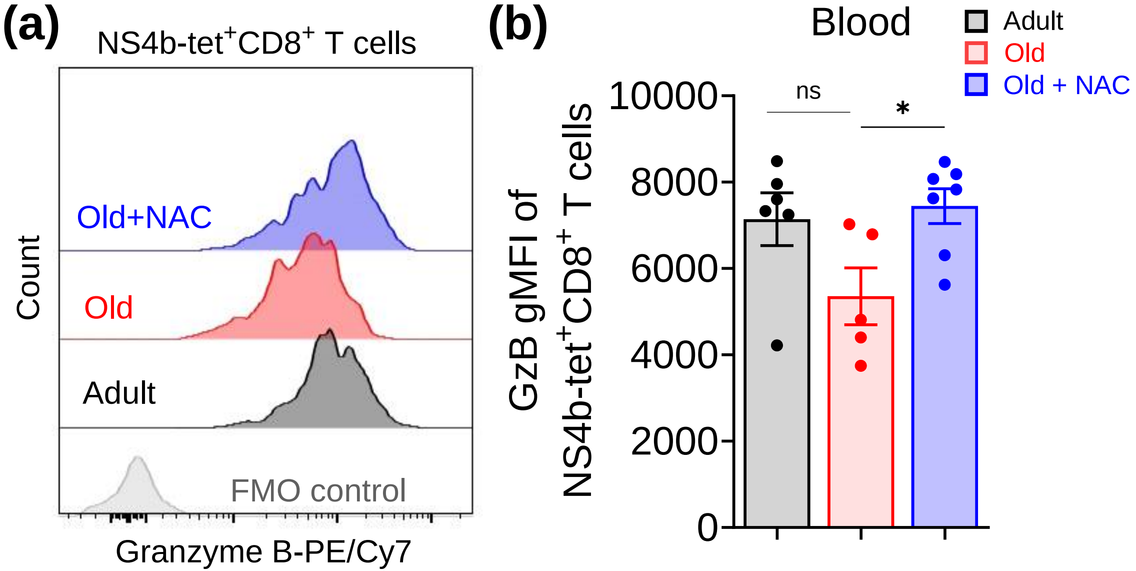
(a)
NS4b-tet+CD8+ T cells
Old+NAC
Old
Adult
FMO control
Count
Granzyme B-PE/Cy7
(b)
Blood
Adult
Old
Old + NAC
ns
*
GzB gMFI of NS4b-tet+CD8+ T cells
10000
8000
6000
4000
2000
0

Figure S10

Figure S10. N-Acetyl Cysteine treatment augments the cytotoxic capacity of West Nile virus-specific T cells. Old (18-20 mo) C57BL/6 male mice treated with N-Acetyl Cysteine (NAC) every alternate day by oral gavage for 4 continuous weeks, followed by s.c. infection with WNV in both hind foot-pads. At 7 d.p.i., WNV-specific T cell response was analyzed. **(a)** Representative flow cytometry plots (gated on live, $CD3^+CD4^-CD8^+$H-2D(b)-NS4b-tetramer$^+$ cells) show intracellular granzyme B expression in blood from adult, old, and NAC-treated old mice. Fluorescence minus one (FMO) control shows the background fluorescence. **(b)** Data show geometric MFI of granzyme B in H-2D(b)-NS4b-tetramer$^+$$CD8^+$ T cells. Data is representative of 2 independent experiments and expressed as mean ± s.e.m. Each dot represents an individual mouse. One-way ANOVA followed by Tukey's multiple comparison test (b). ns- non-significant, * $p \leq 0.05$, ** $p \leq 0.01$, *** $p \leq 0.001$, and **** $p \leq 0.0001$.

Table S1. List of antibodies and reagents.

**1. Antibodies**		
**Reagents**	**Source**	**Identifier**
Anti-mouse CD3-PerCP-Cy5.5 (clone 17A2)	Biolegend	Cat no. 100218
Anti-mouse CD3-FITC (clone 17A2)	Biolegend	Cat no. 100203
Anti-mouse CD4-Brilliant Violet 750 (clone GK1.5)	Biolegend	Cat no. 100467
Anti-mouse CD8-Brilliant Violet 785 (clone 53-6.7)	Biolegend	Cat no. 100750
Anti-mouseCD8b-PerCP/Cy5.5 (clone YTS156.7.7)	Biolegend	Cat no. 126609
Anti-mouse CD62L-APC-Cy7 (clone MEL-14)	Biolegend	Cat no. 104428
Anti-mouse CD62L-APC-Fire 750 (clone MEL-14)	Biolegend	Cat no. 104450
Anti-mouse CD62L-PE/Dazzle 594 (clone MEL-14)	Biolegend	Cat no. 104448
Anti-human/mouse CD44-Brilliant Violet 570 (clone IM7)	Biolegend	Cat no. 103037
Anti-mouse CD25-PE (clone PC61)	Biolegend	Cat no. 102008
Anti-mouse CD69-PE-Dazzel 594 (clone H1.2F3)	Biolegend	Cat no. 104535
Anti-mouse CD127-PE-Cy7 (clone A7R34)	Biolegend	Cat no. 135014
Anti-mouse CCR7-PE-Cy7 (clone 4B12)	eBioscience	Cat no. 25-1971-82
Anti-human/mouse Granzyme B-PE-Cy7 (clone QA16A02)	Biolegend	Cat no. 372214
Anti-mouse CD45-eFluor 450 (clone 30-F11)	eBioscience	Cat no. 48-0451-80
Anti-mouse CD45-Brilliant Violet 570 (clone 30-F11)	Biolegend	Cat no. 103136
Anti-mouse CD45-Brilliant Violet 650 (clone 30-F11)	Biolegend	Cat no. 103151
Anti-mouse CD45- Alexa Fluor 700 (clone 30-F11)	Biolegend	Cat no. 103128
Anti-mouse Ter-119-eFluor 450 (clone TER-119)	eBioscience	Cat no. 48-5921-82
Anti-mouse Ter-119- Brilliant Violet 650 (clone TER-119)	Biolegend	Cat no. 116235
Anti-mouse Ter-119- Alexa Fluor 700 (clone TER-119)	Biolegend	Cat no. 116220
Anti-mouse CD31-Brilliant Violet 785 (clone 390)	Biolegend	Cat no. 102435
Anti-mouse CD31-PE (clone 390)	Biolegend	Cat no. 102407
Anti-mouse CD31-Alexa Fluor 647 (clone 390)	Biolegend	Cat no. 102415
Anti-mouse Podoplanin-Brilliant Violet 421 (clone 8.1.1)	Biolegend	Cat no. 127423
Anti-mouse Podoplanin-PE (clone 8.1.1)	Biolegend	Cat no. 127408
Anti-mouse Podoplanin-PE-Cy7 (clone 8.1.1)	Biolegend	Cat no. 127412
Anti-mouse CD5-Brilliant Violet 650 (clone 53-7.3)	BD Bioscience	Cat no. 740444
Anti-mouse BCL2-PE-Cy7 (clone 10C4)	eBioscience	Cat no. 25-6992-42
Anti-human/mouse cleaved caspase 3-Alexa Fluor 488 (clone 269518)	R&D Systems	Cat no. IC835G-100
Anti-human/mouse cleaved caspase 3-Alexa Fluor 647 (clone D3E9)	Cell Signaling Technology	Cat no. 9602S
Anti-Human/mouse BAX polyclonal Ab-CoraLite Plus 488	Thermo Fisher Scientific	Cat no. CL488-50599
Agonistic anti-LTbetaR (clone AF-H6)	Biogen	Ref no. 4749-70
True stain FcX^{TM} anti-mouse CD16/32 (clone 93)	Biolegend	Cat no. 101320

**2. Reagents**	**Source**	**Identifier**
PE-conjugated H-2D(b)-NS4b2488-tetramer	Emory NIH tetramer facility	
APC-conjugated Annexin V	Biolegend	Cat no. 640920
Annexin V binding buffer	Biolegend	Cat no. 422201
Zombie Aqua fixable viability dye	Biolegend	Cat no. 423102
Blue fluorescence reactive dye	Invitrogen	Cat no. L34962A
Liberase TL	Sigma	Cat no. 5401020001
DNase-I	Sigma	Cat no. DN25-100mg
Foxp3 /Transcription factor staining buffer set	eBioscience	Cat no. 00-5523-00
CountBright^{TM} Absolute counting Beads, for flow cytometry	Thermo Fisher Scientific	Cat no. C36950
N-Acetyl Cysteine	Thermo Fisher Scientific	Cat no. A15409.14
Mitoquinone	GLP Bio	Cat no. GC30416
Tocris Bioscience^{TM} Urolithin A	Fisher Scientific	Cat no. 67-621-0
RT2 PCR array	Qiagen	
MitoTracker^{TM} Green FM dye	Invitrogen	Cat no. M46750
MitoTracker^{TM} Deep Red FM dye	Invitrogen	Cat no. M46753
MitoSOX^{TM} Red mitochondrial superoxide indicator	Invitrogen	Cat no. M36008
2’,7’-Dichlorodihydrofluorescein diacetate	Millipore Sigma	Cat no. D6883a
